# Artificial Intelligence–Assisted Image Extraction in Neonatal Echocardiography for Congenital Heart Disease Diagnosis in Sub-Saharan Africa: Protocol for Model Development

**DOI:** 10.2196/75270

**Published:** 2025-10-30

**Authors:** Aminkeng Zawuo Leke, Lionel Landry Sop Deffo, Yunkavi Sabastian Wirsiy, Thomas Aldersley, Thomas Day, Andrew P King, Patrick McAllister, Michel N Maboh, John Lawrenson, Cabral Tantchou, Bernhard Kainz, Frank Casey, Raymond Bond, Dewar Finlay, Ngoe Kelson Tchinda, Armstrong Obale, Frunwi Ndeh Mugri, Liesl Zühlke, Helen Dolk

**Affiliations:** 1 Digital Technology and Innovation Hub, Health Research Foundation Buea Buea Cameroon; 2 Division of Paediatric Cardiology, Department of Paediatrics and Child Health, University of Cape Town Cape Town South Africa; 3 School of Biomedical Engineering and Imaging Sciences, King’s College London London United Kingdom; 4 School of Computing, Ulster University Belfast United Kingdom; 5 St. Elizabeth Catholic general hospital Shisong Cardiac centre Kumbo Cameroon; 6 Department of Computing, South Kensington Campus, Imperial College London London United Kingdom; 7 School of Medicine, Ulster University Londonderry United Kingdom; 8 South African Medical Research Council Cape Town South Africa

**Keywords:** AI-assisted echocardiography, congenital heart disease (CHD) screening, neonatal cardiac imaging, Sub-Saharan Africa health care, telemedicine and AI integration

## Abstract

**Background:**

Sub-Saharan Africa (SSA) bears the highest global burden of under-5 mortality, with congenital heart disease (CHD) as a major contributor. Despite advancements in high-income countries, CHD-related mortality in SSA remains largely unchanged due to limited diagnostic capacity and centralized health care. While pulse oximetry aids early detection, confirmation typically relies on echocardiography, a procedure constrained by a shortage of specialized personnel. Artificial intelligence (AI) offers a promising solution to bridge this diagnostic gap.

**Objective:**

This study aims to develop an AI-assisted echocardiography system that enables nonexpert operators, such as nurses, midwives, and medical doctors, to perform basic cardiac ultrasound sweeps on neonates suspected of CHD and extract accurate cardiac images for remote interpretation by a pediatric cardiologist.

**Methods:**

The study will use a 2-phase approach to develop a deep learning model for real-time cardiac view detection in neonatal echocardiography, utilizing data from St. Padre Pio Hospital in Cameroon and the Red Cross War Memorial Children’s Hospital in South Africa to ensure demographic diversity. In phase 1, the model will be pretrained on retrospective data from nearly 500 neonates (0-28 days old). Phase 2 will fine-tune the model using prospective data from 1000 neonates, which include background elements absent in the retrospective dataset, enabling adaptation to local clinical environments. The datasets will consist of short and continuous echocardiographic video clips covering 10 standard cardiac views, as defined by the American Society of Echocardiography. The model architecture will leverage convolutional neural networks and convolutional long short-term memory layers, inspired by the interleaved visual memory framework, which integrates fast and slow feature extractors via a shared temporal memory mechanism. Video preprocessing, annotation with predefined cardiac view codes using Labelbox, and training with TensorFlow and PyTorch will be performed. Reinforcement learning will guide the dynamic use of feature extractors during training. Iterative refinement, informed by clinical input, will ensure that the model effectively distinguishes correct from incorrect views in real time, enhancing its usability in resource-limited settings.

**Results:**

Retrospective data collection for the project began in September 2024, and to date, data from 308 babies have been collected and labeled. In parallel, the initial model framework has been developed and training initiated using a subset of the labeled data. The project is currently in the intensive execution phase, with all objectives progressing in parallel and final results expected within 10 months.

**Conclusions:**

The AI-assisted echocardiography model developed in this project holds promise for improving early CHD diagnosis and care in SSA and other low-resource settings.

**International Registered Report Identifier (IRRID):**

DERR1-10.2196/75270

## Introduction

Sub-Saharan Africa (SSA) is the region of the world with the highest burden of under-5 mortality: 56.7% of the 4.9 million global under-5 deaths in 2022 occurred in SSA [1], despite the region accounting for only one-quarter of births. As progress has been made in tackling infectious diseases in children, the relative burden of other causes of death, including congenital anomalies (CAs), has increased, such that CAs are now the sixth leading cause of under-5 deaths in SSA [1]. The largest contributor to CA, in terms of both prevalence at birth and mortality, is congenital heart disease (CHD). While child mortality associated with CHD has declined—often dramatically—in most high-income countries (HICs), there has been no improvement in most SSA countries [2]. This lack of progress is impeding achievement of Sustainable Development Goal 3.2, a reduction in child mortality, as well as Sustainable Development Goal 3.8, equity of access to quality essential health care services. The challenges of CHD care in SSA are multifactorial. A recent analysis of African pediatric and CHD services showed that only 18 (40%) countries in Africa were able to provide a full CHD service, including cardiac surgery and interventional cardiac catheterization [3]. Moreover, the capacity of these services is low, with inadequate infrastructure and severe shortages of specialized personnel. Indeed, there are only a median of 0.17 (IQR 0.02-0.35) pediatric cardiologists per million population, far below the internationally recommended ratio of 2 per million [3].

This lack of capacity results in late or missed diagnoses, leading to delayed interventions and poorer outcomes [4,5]. The problem is compounded by the centralization of pediatric cardiac facilities in major urban areas, often located hundreds of kilometers from most birthing centers [4]. To address these challenges, interventions that strengthen the capacity for early CHD detection and decentralize care are critical.

Postnatal screening for CHD involves a combination of clinical examination, pulse oximetry screening, and echocardiography. Pulse oximetry screening is a simple, noninvasive test that measures oxygen saturation in a newborn’s blood [6]. Low oxygen levels, or a significant difference between readings in the hand and foot, may indicate critical CHD and prompt further investigation with echocardiography. Despite recent concerns about the suboptimal performance of the device in dark-skinned infants [7,8], pulse oximetry screening is highly recommended and is increasingly being adopted by many low- and middle-income countries (LMICs), including those in SSA [6,9]. However, pulse oximetry results are not specific to CHD, as low oxygen saturation may also indicate other conditions, such as pneumonia—the leading cause of neonatal mortality in SSA. Therefore, all suspected CHD cases identified by pulse oximetry must be referred to an expert pediatric cardiologist for confirmation through echocardiography. Consequently, the growing uptake of pulse oximetry in SSA is increasing the number of suspected CHD referrals and adding to the workload of the already strained workforce of echocardiographers and pediatric cardiologists. This burden is further compounded by the costs involved and the arduous, often long journeys required for particularly fragile neonates—some of whom may ultimately turn out to be false-positive cases.

The introduction of low-cost, compact mobile ultrasound devices has dramatically increased the availability of ultrasound services in LMICs and has helped to address, to some extent, the centralization of diagnostic services [10]. However, the lack of diagnostic expertise continues to limit their use for highly specialized procedures such as echocardiography. Obtaining optimal echocardiographic images presents significant challenges [10]. The probe must be correctly positioned, and parameters such as gain, contrast, resolution, depth, and magnification must be individually adjusted for each specific view [11]. When combined with patient-related factors such as uncooperative pediatric behavior and complex CHD anatomy, these challenges result in longer scan times, increased workloads, and a greater need for highly trained sonographers or echocardiographers.

Training programs have been shown to improve both image capture and anomaly detection [11]. However, these programs are often labor-intensive and time-consuming, and require repetition due to staff turnover [11]. Therefore, a complementary approach is needed to sustain and build upon the improvements achieved through training.

In recent years, there has been a growing number of projects using artificial intelligence (AI) models to facilitate CHD diagnosis [11-15]. However, most of these projects originate from HICs, potentially limiting their applicability in LMICs due to domain shifts related to equipment, operator expertise, ethnicity, disease profiles, and differences in clinical workflows or infrastructure. Moreover, existing research disproportionately focuses on adult echocardiography, with limited attention to prenatal CHD and little consideration of neonatal applications. Critically, current AI efforts concentrate on image interpretation rather than supporting nonexperts with real-time image acquisition.

**Figure 1:**
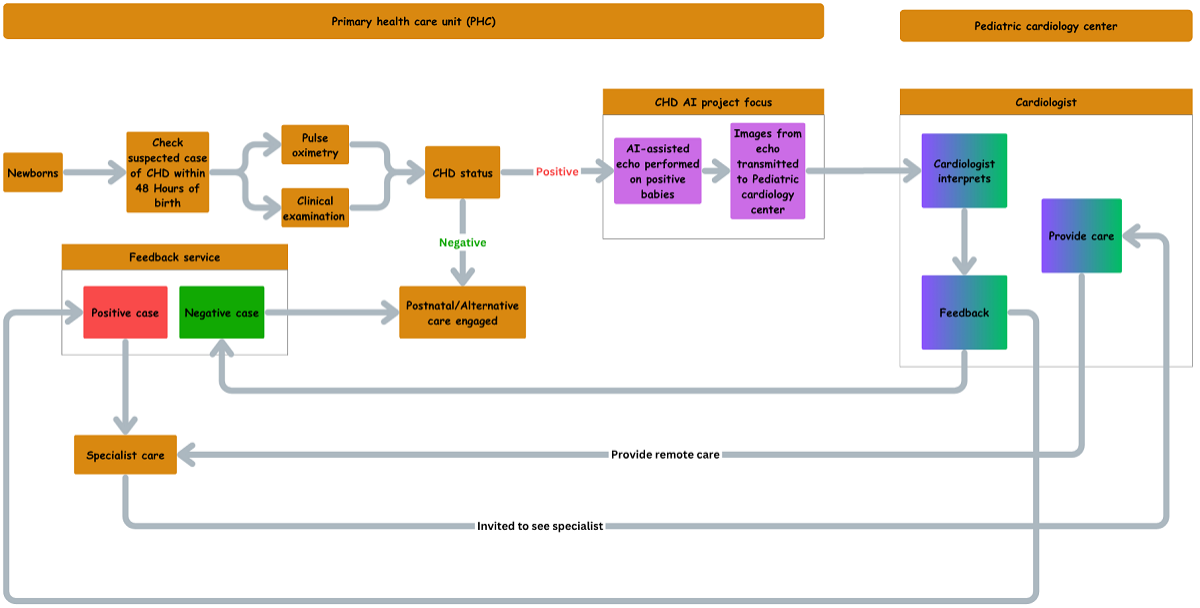
Artificial intelligence (AI)–assisted echocardiography congenital heart disease (CHD) screening process. Echo: echocardiography.

This paper outlines an ongoing project leveraging AI to enable nonexpert echocardiographers—such as sonographers, nurses, midwives, and doctors with minimal specialized training—to perform basic cardiac ultrasound “sweeps” on neonates (aged 0-28 days). The AI system will be designed to automatically extract predefined cardiac views, which can then be transmitted to remote pediatric cardiologists for interpretation. The AI model will be limited to image extraction and will not extend to diagnosing or identifying potential CHD. This approach aims to streamline the initial screening process for CHD. AI-assisted echocardiography will be selectively used for babies with positive pulse oximetry screening results or other clinical indications suggestive of cardiac issues, helping to identify cases requiring further evaluation by specialized pediatric cardiologists (Figure 1).

## Methods

### Study Population and Cardiac Views

This study focuses on extracting accurate cardiac views in neonates aged 0-28 days suspected of CHD. To guide the selection of relevant views, 2 critical questions were considered: (1) can nonexperts reliably maneuver the probe to allow the AI system to capture the desired views, and (2) which views would provide the most valuable insights for cardiologists in determining the need for further investigation. Based on these considerations, and in accordance with the 2024 American Society of Echocardiography guidelines [16], our panel of expert cardiologists identified 10 essential cardiac views (Table 1).

**Table 1:** Selected cardiac views of interest and label codes.

Cardiac view	Label code
1. Subcostal long axis	SCLA
2. Apical 4 chamber	A4C
3. Apical 5 chamber	A5C
4. Parasternal long axis	PLAX
5. Parasternal short axis—aortic valve	PSAX_AV
6. Parasternal short axis—mitral valve	PSAX_MV
7. Parasternal short axis—papillary muscle	PSAX_PM
8. Suprasternal long axis	SSLAX
9. Other diagnostic	OTHER_DIAG
10. Background	BKG

### Data Requirements

To train AI models that guide nonexpert operators in acquiring specific cardiac views, a dataset of real patient ultrasound videos is essential. This dataset must include not only the desired (positive) views but also a substantial amount of background (negative) data representing nonessential or incorrect views. Such data are crucial for enabling the model to effectively distinguish target views from the more common, nonideal images that nonexpert operators are likely to produce in real-world settings.

Existing data saved by expert cardiologists during routine clinical scans provide a readily available source with minimal ethical concerns. However, cardiologists typically save only the most relevant cardiac views, meaning that existing hospital datasets contain these specific views without the necessary background elements. Additionally, a significant domain shift exists between existing datasets—primarily collected in HICs—and newly acquired data from LMICs, where variations in clinical protocols, imaging equipment, and patient demographics may affect model generalizability. Furthermore, the scarcity of publicly available neonatal echocardiography datasets makes data acquisition a critical challenge. To obtain data containing both target views and background elements, new data must be collected prospectively. This approach also allows for modifications to facilitate frame extraction and labeling, as described in the “Data Labeling” section. However, prospective data collection is time-consuming and resource-intensive. Ethical considerations must also be addressed, including obtaining informed consent from parents and ensuring that data collection does not disrupt routine clinical workflows. A practical approach to mitigate these limitations while maximizing the benefits of both dataset types is to develop the model in 2 phases: initially leveraging existing hospital datasets for pretraining, followed by fine-tuning with prospectively collected data to ensure adaptability to LMIC settings.

In the first phase, existing data will be used to build an initial model framework, while prospective data are collected simultaneously. Once the new prospective data become available, they will be used in the second phase to refine and enhance the initial model developed from the existing data (see the “Model Building” section). This hybrid approach enables the project to leverage readily available existing data to initiate model development, while ensuring that the final model is trained on a comprehensive dataset containing both the target cardiac views and the necessary background elements. Both types of datasets will include all scanned cases, regardless of whether the neonate’s heart was abnormal.

The proposed AI model is intended for use in neonates aged 0-28 days. However, to expedite data collection and increase the sample size, the project will also include data from infants aged 0-2 years, based on the observation that the cardiac structure of older infants is similar to that of neonates [17].

In the first phase of model development, the project will aim to gather short anonymized echocardiographic video clips from 500 babies. Each dataset will consist of 1-7 clips per baby, depending on scan availability and quality. These clips are expected to include 1 or more of the 10 cardiac views utilized in the project. The retrospective dataset of 500 babies will be selected to provide a minimum of 1500-3500 labeled echocardiographic clips, ensuring at least 73 examples per cardiac view to statistically support a 95% accuracy target with a 5% margin of error at 95% confidence. Each video clip is expected to be approximately 3-5 seconds long, with an average file size of up to 4 MB. Based on these parameters, the total number of clips is estimated to range from 1500 to 3500, resulting in an expected storage requirement of approximately 14 GB for the raw video data. Additional space for data preprocessing, model training, and backups will increase the total storage needed for this phase to around 200 GB, including 50 GB allocated for backups.

In the second phase of model development, the project will collect prospective echocardiographic video data from 1000 babies. Each baby will have 1 continuous video clip representing a complete echocardiographic scan, recorded at a higher frame rate of 60 frames/second, with an average duration of 20 minutes. Shorter video clips corresponding to individual cardiac views will subsequently be extracted from these long recordings for analysis. The sample of 1000 babies has been chosen to ensure sufficient diversity in anatomical presentations, imaging conditions, and potential CHDs, enabling the development of a robust and generalizable model applicable across varied clinical contexts. In addition to this contextual rationale, the sample size will provide sufficient statistical power to support model development. Assuming a minimum of 203 labeled examples per cardiac view is required to achieve 95% classification accuracy with a 3% margin of error at 95% confidence, the sample of 1000 babies is expected to yield the necessary number of view-specific clips to meet this threshold, accounting for natural variation in view representation. Each 20-minute video is anticipated to have an average file size of 1 GB, resulting in a total raw data size of approximately 1 TB. Accounting for data preprocessing, extraction of shorter clips, model training, and storage redundancy, the total storage requirement for this phase is projected to reach around 15 TB, including 5 TB allocated for backups.

By focusing on video clips directly, the project will simplify data collection while preserving the variability and dynamic features necessary for robust model training. The shorter clips extracted in the second phase will ensure compatibility with the analysis framework established in the first phase.

### Study Sites and Data Collection

The project will collect data from 2 hospitals: St. Padre Pio Hospital in Douala, Cameroon, and the Red Cross War Memorial Children’s Hospital in Cape Town, South Africa. Approximately 80% of the data will come from South Africa, with Cameroon contributing 20%. These sites were carefully selected to ensure representation of diverse patient demographics, which will be critical for the performance and external validity of the developed model. Data from both sites will be pooled and randomly split for training, validation, and testing. While the model does not use a dedicated external dataset, the inclusion of diverse populations and imaging conditions is expected to enhance its generalizability across clinical contexts in SSA. This approach allows the inclusion of data from multiple facilities using different ultrasound machines. Consequently, metadata on equipment type, settings, and resolution will be collected to assess potential heterogeneity. Where possible, domain adaptation techniques will be explored to further improve model robustness.

Cameroon is a bilingual (French and English) country in central Africa, often referred to as “Africa in Miniature.” The country reflects the vast diversity of SSA, encompassing multiple cultural, linguistic, and geographical variations. St. Padre Pio Hospital in Douala is one of the few referral centers for pediatric cardiology in Cameroon and receives patients from across the country. The Children’s Heart Disease Research Unit at the Red Cross War Memorial Children’s Hospital in Cape Town serves a racially and ethnically diverse population, with considerable variability in socioeconomic factors and access to health care. Together, data from Cameroon and South Africa will provide the representativeness needed to ensure that the model performs effectively across different clinical conditions and patient demographics in SSA.

Existing data (short video clips) will be extracted from locally saved sources at the participating hospitals. Prospective data will be collected during routine echocardiography scans performed by expert cardiologists or sonographers. Before data collection, the experts will obtain informed consent from the parents or legal guardians of each participating infant. The informed consent form and study information sheet are provided in Multimedia Appendix 1. Parents may refuse participation without any impact on the care their child receives. The project team is committed to conducting data collection ethically, respecting the rights and well-being of participating families. It is essential that the process does not interfere with routine clinical care. The expert is expected to perform the scan as usual, with 2 simple modifications:

- Before starting the scan, the expert shall connect a screen-capture device to record the entire echocardiography session from start to finish.
- For the cardiac views of interest, in addition to the usual routine pause to save the frame, the expert is expected to label the frame at a defined position (top-right corner of the screen).

At the end of the scan, the recorded video will be saved on a separate screen-capture device. A standard operating procedure (SOP) has been developed to guide all cardiologists involved in the prospective data collection, minimizing operator variability (Multimedia Appendix 2). Our expert cardiologists believe that labeling the frames during the scan will not affect the typical scan duration.

### Data Deidentification, Metadata Generation, and Data Transmission and Storage

#### Overview

This project is part of the broader Data Science for Health Discovery and Innovation in Africa (DS-I Africa) Network. As one of the network’s research initiatives, it will leverage the on-premises cloud storage and computing infrastructure provided by DS-I Africa through its eLwazi Open Data Science Platform. This infrastructure, specifically the **Illifu system**, forms the backbone of the data transmission and storage processes described in the “**Data Transmission and Storage”** section, enabling secure and efficient data management.

All existing and prospective data will be transmitted to a central, secure on-premises cloud storage solution, where model building and data analysis will be conducted. Before transmission, the data will undergo several processes, including deidentification (covering both anonymization and pseudonymization), metadata generation, and secure transfer. These processes will ensure that data privacy, integrity, and security are maintained, adhering to the highest standards of data protection.

**Figure 2:**
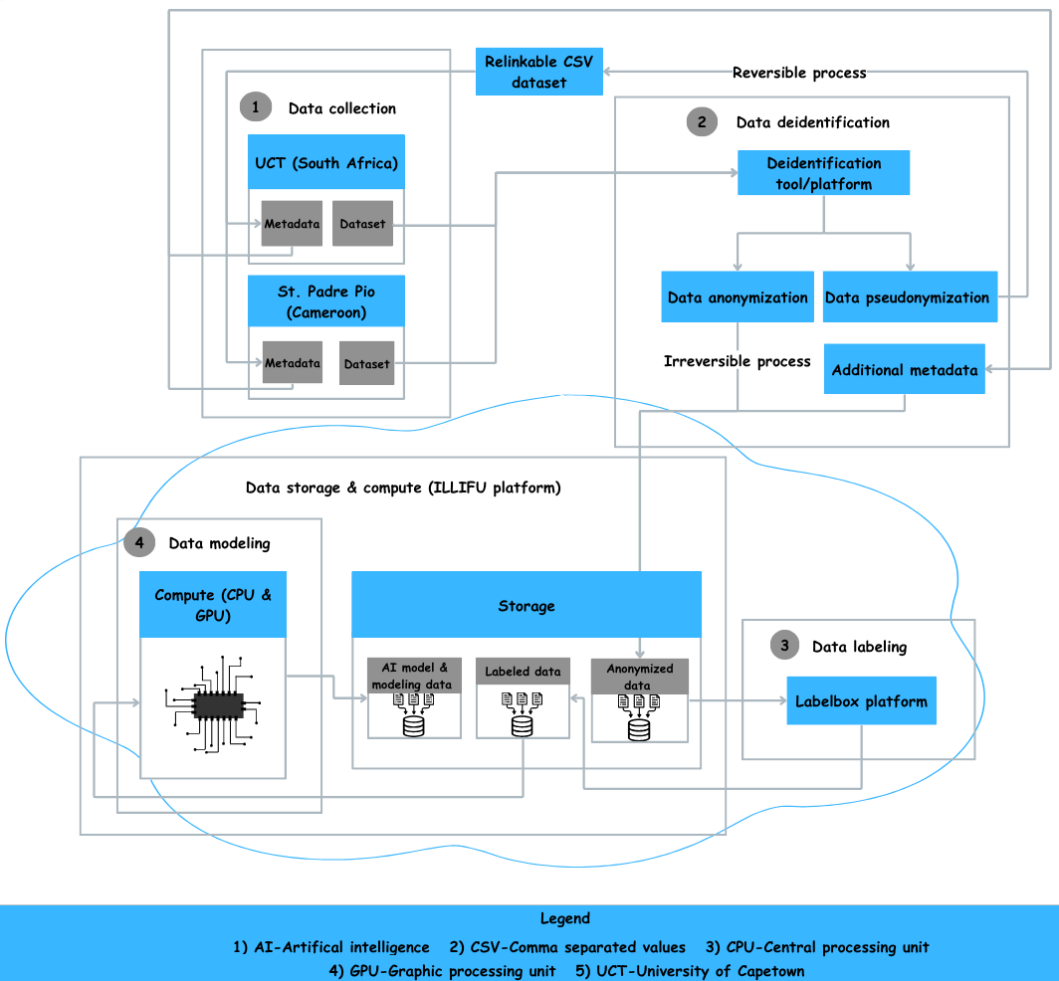
Thematic data flow diagram for the CHD-AI project. AI: artificial intelligence; CHD: congenital heart disease.

The thematic diagram (Figure 2) illustrates the overall process flow, including data collection, deidentification, metadata generation, and secure transmission and storage. It provides a visual overview of how data progresses through each stage of the workflow.

#### Data Deidentification

To ensure patient privacy and compliance with ethical guidelines, all data will undergo a deidentification process to remove personally identifiable information before use. This will be implemented using a custom anonymization script developed by the project engineering team, which automates the removal of sensitive details while preserving data integrity. Additionally, unique project identification codes will be assigned to facilitate internal traceability without revealing personal information. When controlled data access is required for specific research purposes, a secure pseudonymization approach will be used, allowing authorized personnel to reference anonymized data without compromising privacy.

#### Metadata Generation

After deidentification, metadata are generated to provide contextual information about the data, as shown in Figure 2. These metadata will support subsequent data management and analysis.

- Types of metadata: Includes patient age, sex, and diagnosis to support analysis and traceability.
- Automated metadata extraction: Ensures consistency and minimizes human error by generating metadata automatically using a script developed by the engineering team.
- Use of metadata: Metadata will accompany anonymized and pseudonymized data as needed, maintaining transparency and traceability while adhering to privacy requirements.

#### Data Transmission and Storage

For this study, data will be securely transmitted to the Illifu on-premises cloud storage solution provided by the eLwazi Open Data Science Platform, a key infrastructure component of the DS-I Africa Network. This stage, as illustrated in Figure 2, ensures secure and compliant storage accessible only to authorized personnel. The platform will also serve as the computing environment for model development, providing the necessary resources for data processing and analysis.

The decision to utilize the Illifu infrastructure reflects its strong alignment with DS-I Africa’s goals of advancing data science capabilities across its projects. While Illifu serves as the primary infrastructure, similar objectives could be achieved using other cloud storage solutions—such as Google Cloud Platform, Amazon Web Services, or Microsoft Azure—provided they are configured to meet required privacy and compliance standards.

### Data Labeling

#### Existing Data Labeling

Labeling of the existing echocardiographic data will be conducted by expert cardiologists using the Labelbox platform, integrated with Google Cloud. This process will ensure consistent, high-quality annotations essential for model training (Textbox 1).

**Labeling procedure.**

**1. Labeling parameters**

The labeling will involve the following parameters:

- Cardiac views: Each video clip will be assigned 1 of the 10 predefined cardiac views, as determined by the expert cardiologists in the project and for each view, a subparameter will indicate the presence of color Doppler, labeled as “Color” or “No Color” depending on whether color Doppler imaging is visible in the clip.
- Frame quality: Each frame will be rated for quality on a 3-tier scale: poor, normal, or good. This rating will help filter and prioritize frames for model training.
- Metadata integration: Labels will be linked to metadata, including patient-specific information (age, sex, and diagnosis) and labeling-specific attributes (labeling cardiologist ID and time stamp).

**2. Labeling workload and quality assurance**

As the labeling process is demanding, each video clip will be labeled by a single cardiologist. To ensure interrater reliability, 5% of each cardiologist’s labeled data will be randomly assigned to a second cardiologist for cross-validation and assessment of variability.

We anticipate the participation of 10 cardiologists, with each assigned approximately 500 video clips to label over a 3-month period.

#### Prospective Data Labeling: Automated Label Derivation

Prospective data collected during routine echocardiography scans already include labels of the cardiac views assigned by the cardiologist or sonographer. However, these labels cannot be directly used to train the AI model, as the model would merely learn to recognize the labels rather than understanding the actual cardiac views. This would limit its ability to generalize and identify unlabeled cardiac views in real-world applications.

To address this challenge, the team will develop an automated script to extract unlabeled cardiac views from the labeled prospective data. The script will analyze the ultrasound video clips, identifying pauses that indicate when the expert saved specific views. It will then assign the names of these saved views based on the expert-assigned labels. Finally, the script will extract unlabeled video segments from between these pauses, representing the same cardiac views labeled by the expert but without visible labels.

The unlabeled clips, along with their associated labels stored separately, will then be uploaded into Labelbox for further annotation of video quality and any additional view-specific parameters. The aim is to provide diverse examples of the same cardiac view for model training while keeping the labels hidden during the training phase.

To ensure consistency in labeling, a subset of the labeled data will be independently reviewed by multiple annotators, and interrater reliability will be assessed using the Cohen κ statistic. Any discrepancies will be resolved by consensus or by a senior clinical reviewer.

### Metadata Extraction and Format

**Figure 3:**
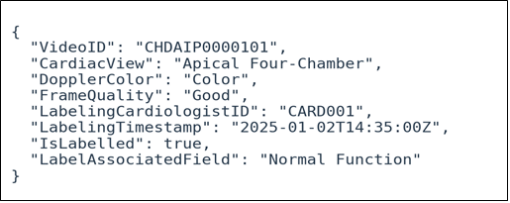
Sample of labeled video clip metadata structure in JSON format.

After labeling, the data will be exported in JSON format from the Labelbox platform and processed for model training in the Python (Python Foundation) environment provided by the Illifu platform. The metadata will follow a predefined structure to ensure consistency and usability across the dataset. This structure will include the following key details: Video ID, Cardiac View, Doppler Color, Frame Quality, Labeling Cardiologist ID, Labeling Timestamp, Is Labeled, and Label-Associated Field. Figure 3 illustrates the standardized format for labeled video metadata, ensuring consistency and usability for model training. This structured format allows labeled and unlabeled data to be handled separately while maintaining consistent metadata attributes. It also facilitates tracking and integration of the data into the model training pipeline.

Every labeled video exported from Labelbox will follow this predefined format, ensuring uniformity and ease of integration. Each video will be uniquely identified by a “Video ID,” such as CHDAIP0000101. The labels of interest extracted from the labeled dataset are Cardiac View, Frame Quality, and Doppler Color. The “Cardiac View” parameter specifies the view represented in the video, with the available options detailed in Table 1. The “Doppler Color” parameter indicates whether color Doppler imaging is present, labeled as either “Color” or “No Color.” The overall quality of the video frames is assessed under the “Frame Quality” parameter, categorized as “good,” “medium,” or “poor” based on clarity and diagnostic usability. Each video is also associated with a “Labeling Cardiologist ID,” serving as a unique identifier for the cardiologist responsible for labeling, along with a “Labeling Timestamp” recording the exact date and time of labeling. The “Is Labelled” parameter distinguishes between frames that are prospectively labeled and those extracted by the automated script. For unlabeled clips, the “Label Associated Field” will contain labels derived from the automated script, ensuring that these clips can still be used in analysis.

**Figure 4:**
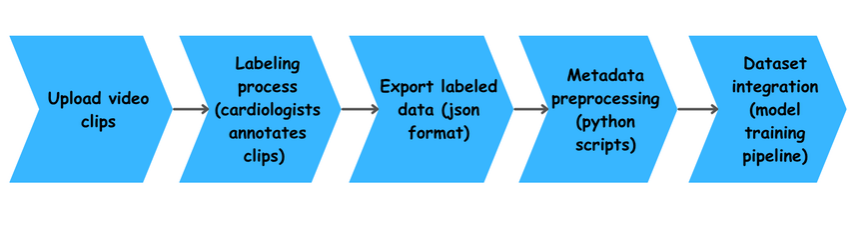
Metadata processing steps.

This structured format will not only ensure consistency in metadata attributes but also enable efficient tracking of labeled and unlabeled data. It will streamline the integration of these data points into the model training pipeline, enhancing the overall accuracy and reliability of the process. The flowchart in Figure 4 illustrates the entire workflow.

### Development of the AI Model

#### Methodology and Deployment Considerations

The proposed AI model will build upon the successful and validated work of Gearhart et al [18] and Mason et al [19]. Its development will involve 4 key steps: data collection and preparation, data preprocessing, data exploration and visualization, and model building and evaluation.

Although transformer-based architectures represent a promising area of research in computer vision, our focus will be on solutions that can be feasibly deployed in remote, resource-constrained environments. Therefore, we will prioritize lightweight and computationally efficient convolutional neural network models, such as MobileNet and EfficientNet, which are better suited for real-time inference on low-power devices.

#### Data Preprocessing

Data processing will involve 2 major tasks: (1) enhancement of pictorial information for human interpretation and (2) processing of image data for storage, transmission, and representation for autonomous machine perception. The data preprocessing steps can be categorized into low-, mid-, and high-level processes (Textbox 2).

**Data preprocessing steps.**

**1. Low-level processes**

Images and frames extracted from videos will undergo basic image processing operations, such as Gaussian filtering for noise reduction, histogram equalization for contrast enhancement, and unsharp masking for image sharpening. These steps improve the clarity and quality of input images before further processing.

**2. Mid-level processes**

Following the procedure described in Gearhart et al [18], images from the low-level processing step will undergo further processing, including removing Doppler and split views (segmentation) and sampling random background frames.

**3. High-level processes**

Images from the mid-level processing step will undergo further processing, including cropping the field of view and rescaling, inpainting labels and annotations on the images, and splitting the data into training and test sets.

#### Data Exploration and Visualization

This step will involve identifying common data issues that may arise during model training, some of which relate to object detection, as we will be detecting the cardiac plane and heart structures. We anticipate issues related to the following:

- Image dimensions and aspect ratios: Images may have different sizes (eg, one image could be 32×32, while another could be 123×47).
- Label composition: Imbalances in labels, including variations in bounding box sizes and aspect ratios, may occur (eg, some cardiac views may have more images than others).

To address these issues, we will use dedicated tools such as Google Facets (Google LLC/Alphabet Inc), a data visualization platform, to explore the image data and make informed decisions accordingly.

#### Model Building

#### Phase 1: Model Development

To detect cardiac views and localize heart structures in newborns using real-time echocardiography videos, we will adopt a novel approach inspired by Gearhart et al [18]. Their work demonstrated an efficient pipeline for video object detection, achieving state-of-the-art performance.

To adapt their architecture for our dataset, we propose an interleaved framework with 2 feature extractors of distinct speeds and recognition capabilities. These extractors will process different frames, with the extracted features maintained in a shared visual memory through a convolutional long short-term memory layer. The convolutional long short-term memory fuses contextual information from previous frames with features from the current frame to generate accurate detections.

Following Gearhart et al [18]’s approach, the interleaving policy, which determines when to run each feature extractor, will be learned through reinforcement learning. The convolutional long short-term memory is particularly well-suited for sequential image data, making it ideal for analyzing short echocardiography video clips. The training specifics are presented in Textbox 3.

**Training specifics.**

**Dataset composition**

The dataset consists of short echocardiography video clips in AVI format (St. Padre Pio Clinic, Cameroon) and DICOM format (University of Cape Town, South Africa), along with metadata files in Excel (Microsoft Corp) format containing patient age, sex, and diagnosis.

**Metadata utilization**

Metadata will be incorporated into model training to enhance feature representation, enabling the model to leverage diagnostic context alongside visual data.

**Labeling annotations**

Video clips will be annotated on the Labelbox platform with the following:

- Cardiac view: 10 predefined categories, with subparameters indicating Doppler presence.
- Video quality: categorized as poor, normal, or good.

**Figure 5:**
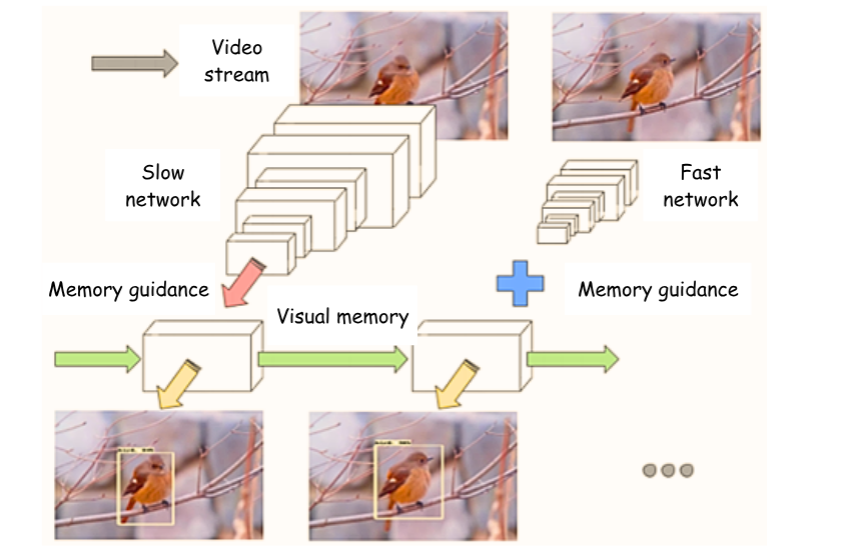
An illustration of a memory-guided interleaved model.

As illustrated in Figure 5, given a video stream, a visual memory model fuses features from fast and slow feature extractors across frames to generate live detections. The layers and weights of this pretrained model will be fine-tuned on our dataset, focusing specifically on the cardiac views of newborns.

It is important to emphasize that, to mitigate overfitting, training will incorporate early stopping, dropout regularization, and k-fold cross-validation. If signs of overfitting are observed during training (eg, a large divergence between training and validation loss), the model complexity will be adjusted accordingly. In addition to convolutional neural networks, we will explore transformer-based architectures, which have shown promise in video and sequential image interpretation tasks. Comparative performance evaluations will be conducted to determine their suitability in this clinical context.

#### Training Process

Training will follow an iterative approach, with checkpoints saved at regular intervals to allow seamless resumption in case of interruptions. The model will initially be trained from scratch using the collected and constructed dataset. As additional data become available from various sources, a fine-tuning strategy will be employed to gradually enhance performance and adapt the model to new variations. Training will be conducted using libraries such as TensorFlow and PyTorch, leveraging high-performance graphical processing units on the computing cluster to optimize efficiency and scalability.

#### Phase 2: Model Refinement

In phase 2, prospective data will be used to refine the initial model, ensuring improved accuracy and adaptability. The model will leverage prediction confidence (PC) to optimize video clip extraction and assess quality (Textbox 4).

**Model refinement.**

**1. Dynamic prediction confidence**

Given an ultrasound video stream, the model will compute the probability (prediction confidence [PC]) that an extracted image frame or video clip corresponds to a specific cardiac view:

- Video clip extraction will begin once a defined PC threshold is met for an image frame.
- Subsequent frames meeting the PC threshold will be compiled into a 5-second video clip for the identified view.
- If multiple video clips of the same view are generated, the clip with the highest average PC across frames will be retained as the final clip.

**2. Video quality optimization**

As image quality can vary depending on the ultrasound device, the model will allow users to set a PC threshold during first use to ensure optimal image quality:

- High PC: If the initial PC setting exceeds the device’s capabilities, the model will prompt the user to lower the threshold.
- Low PC: Conversely, if the initial PC setting is too low, the model will suggest increasing the threshold to maximize quality.

These mechanisms will ensure that the highest-quality images are extracted, regardless of device variability. An accompanying manual will guide users to start with higher PC settings during initial model use.

**3. Integration with training pipeline**

- During model refinement, metadata (eg, age, sex, diagnosis) will be used alongside visual data to enhance feature representation and improve diagnostic accuracy.
- Labels generated during the annotation phase (eg, cardiac view, Doppler presence, and video quality) will also be incorporated into the refinement process.

**Figure 6:**
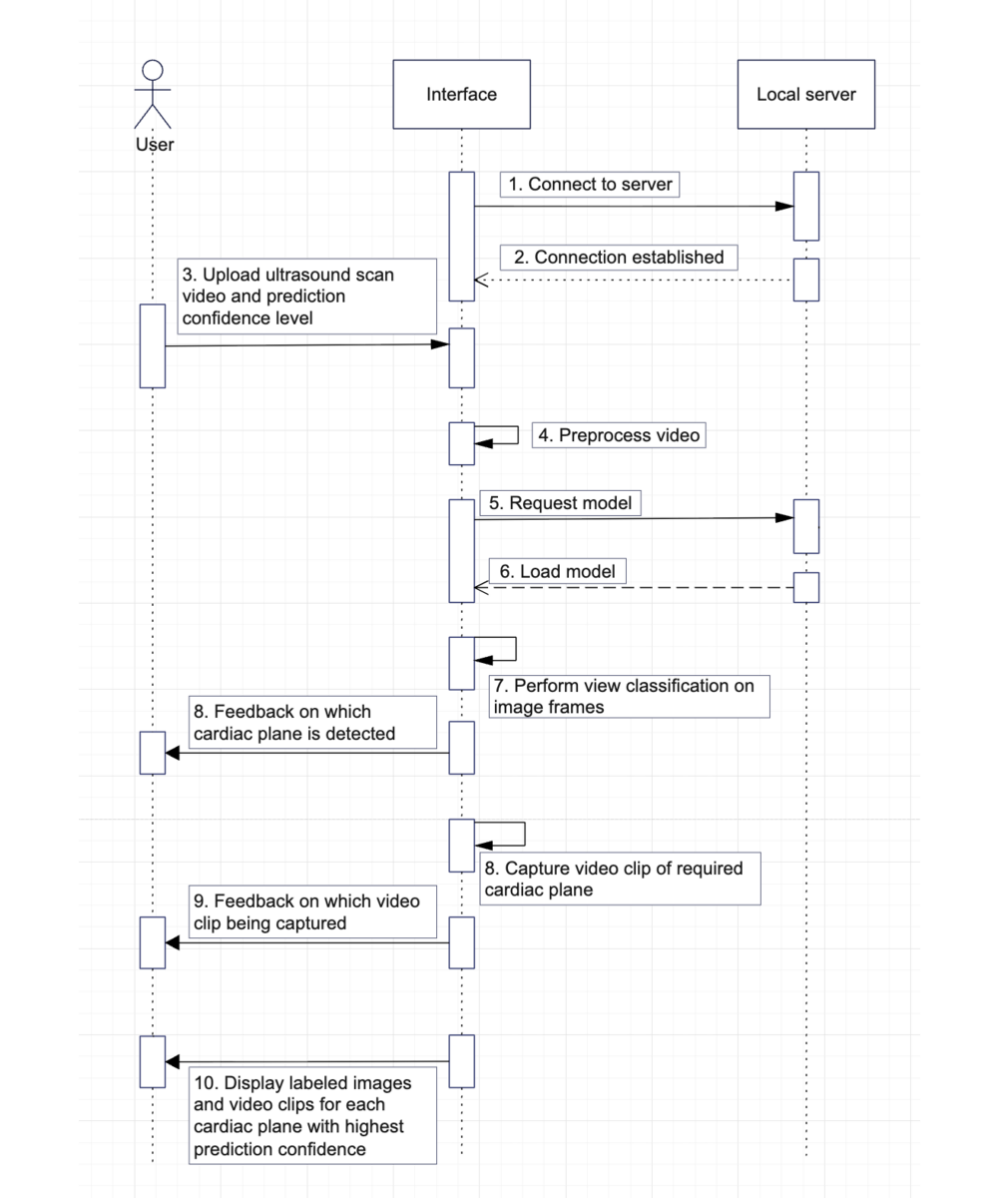
Sequence diagram of our artificial intelligence model system.

**Figure 7:**
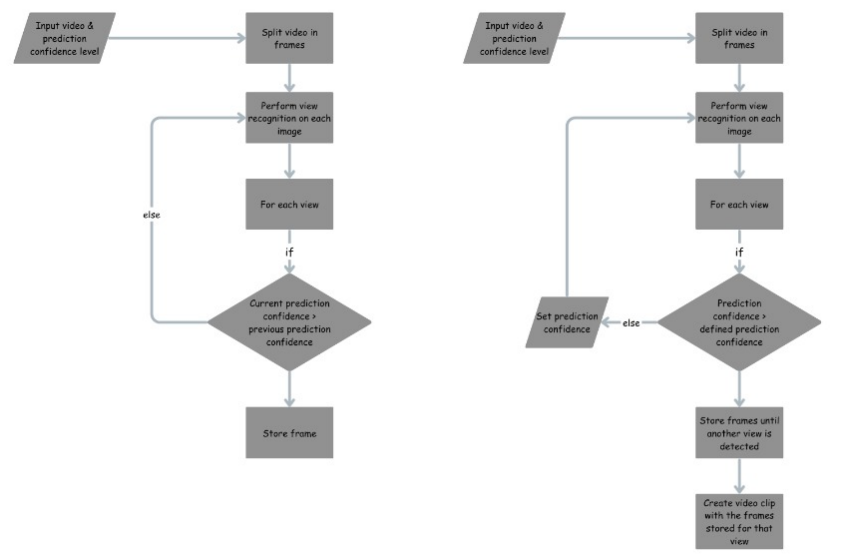
Extractions of sequence diagram.

Figures 6 and 7 illustrate the sequence and flow of the video clip extraction and PC adjustment mechanisms during real-time ultrasound scans.

**4. Refinement goals**

- To integrate metadata-driven learning by leveraging age, sex, and diagnosis to fine-tune the model.
- To enhance adaptability across different ultrasound devices while ensuring robust performance.

**5. Expected outcomes**

The proposed protocol combines state-of-the-art video object detection with metadata integration, enabling robust detection of cardiac views and localization of heart structures in echocardiography video streams. The model’s reinforcement learning–based interleaving policies, dynamic PC adjustments, and quality control mechanisms ensure adaptability across different devices and datasets.

#### Model Evaluation

To comprehensively assess the model’s performance in detecting cardiac views and localizing heart structures in neonates, a robust evaluation framework will be implemented. The evaluation will use an 80/20 training-testing split to ensure a representative data distribution. Additionally, k-fold cross-validation (eg, 10-fold) will be applied to improve the reliability of performance metrics by averaging results across multiple splits. The model will be trained, validated, and tested on a combined dataset from Cameroon and South Africa using stratified random splits. Although no site is reserved exclusively for external validation, this approach is designed to capture variability across regions and equipment, supporting real-world generalizability. The evaluation metrics are listed in Textbox 5.

**Evaluation metrics.**

**1. Overall accuracy**

The percentage of correctly classified instances across all classes in the test dataset.

**2. Per-class accuracy**

The accuracy for each individual cardiac view, providing insights into the model’s performance across different heart structures.

**3. Average accuracy**

The mean accuracy across all classes, offering a balanced assessment of class-specific predictions.

**4. Confusion matrix analysis**

Confusion matrices will be generated to visualize true positives, false positives, true negatives, and false negatives for each class. These matrices will help identify systematic misclassifications and underperforming classes.

**5. F1-score**

The harmonic mean of precision and recall, calculated for each class, to measure the balance between false positives and false negatives. The macro *F*_1_-score will summarize overall classification performance.

**6. Receiver operating characteristic curve and area under the curve**

For binary classification tasks (eg, Doppler vs non-Doppler views), receiver operating characteristic curves will be plotted to illustrate the trade-off between sensitivity and specificity. The area under the curve will quantify the model’s discriminatory ability.

**7. C-statistics**

Calculated to measure the concordance between predicted and actual classes, serving as an overall indicator of the model’s diagnostic performance.

**8. Saliency maps**

- Visualization techniques will be applied to highlight regions in input images that strongly influence the model’s predictions, enhancing interpretability and ensuring alignment with domain knowledge, such as focusing on heart structures in echocardiography images.
- To support explainable artificial intelligence, methods such as Grad-CAM, Grad-CAM++, and other class activation mapping techniques will be utilized.
- These approaches will help visualize the model’s decision-making process, providing deeper insights into how specific features contribute to classification outcomes.

**9. Class-specific evaluation metrics**

- Precision (positive predictive value): The fraction of correctly predicted positive observations.
- Recall (sensitivity): The ability of the model to identify all relevant instances in a class.
- Specificity: The ability of the model to exclude irrelevant instances.

**10. Video-based metrics**

For evaluating performance on the short video clips, the following will be evaluated:

- Clip prediction accuracy: The percentage of correctly classified video clips, where the assigned view corresponds to the majority or highest-confidence prediction across frames.
- Temporal consistency: The consistency of predictions within a video clip, measured as the proportion of consecutive frames with matching predictions.

**11. Metadata-driven analysis**

Using age, sex, and diagnosis from the metadata, model performance will be evaluated for specific subpopulations (eg, neonates with congenital heart disease vs those without). This ensures that the model performs robustly across diverse patient demographics and clinical conditions.

By combining these quantitative and qualitative evaluation metrics, the model’s accuracy, reliability, and interpretability will be rigorously assessed. The evaluation will follow the approach of Mason et al [19] but will be extended to include video-based and metadata-specific metrics, accounting for the unique characteristics of the dataset.

### Model Interpretability and Explainability

In recognition of the importance of trust and transparency in clinical decision-making, the model will incorporate interpretability techniques to support clinician acceptance and regulatory readiness. Specifically, saliency map methods such as Grad-CAM will be applied to highlight image regions that most influence model outputs. These visualizations will be used during internal review and validation phases to confirm alignment with clinically relevant anatomical features. During pilot implementation, these explainability outputs will be presented to end users—pediatric cardiologists and trained frontline clinicians—for interpretability assessment. This process will help ensure that model behavior is understandable, clinically grounded, and suitable for integration into human-in-the-loop diagnostic workflows.

### Workflow

A GitHub repository for the project will be created, and members of the engineering team will have access to clone it to their local machines and contribute code. A branch protection rule will be implemented so that all contributions are reviewed by the administrator (senior engineer) before merging. The repository will also include the work of Gearhart et al [18], which is freely available on GitHub.

### SOP and Instructional Video on Echocardiography Sweep for Nonexperts

Using relevant materials from the literature, our expert cardiologist on the research team will develop a step-by-step procedure for performing an echocardiography sweep, enabling the AI model to capture the defined frames/cardiac views of interest. This procedure is designed to support nonexpert operators in acquiring the correct cardiac views and capturing usable echocardiographic video (see Multimedia Appendix 2). This SOP will be complemented by an instructional video included in the project toolkit. The video will detail preparatory steps before initiating an echo scan, tips on probe handling and maneuvering, and the sequence of moving the probe across the baby’s chest, including start and end positions. Once the SOP is finalized, it will serve as the basis for developing the instructional video to facilitate training of nonexpert operators.

### Investigating the Diagnostic Utility of the Video Clips Produced From the AI Model in Relation to Their Use in Clinical Practice

#### Overview

We will evaluate the clinical utility of the AI-extracted video clips through the following steps.

#### Develop a Platform to Operate the Model

The platform will run on a local computer or server and serve as an interface for the AI model to process uploaded live video streams.

#### Obtain Live-Stream Echocardiography Videos From Clinical Practice

We will recruit 4 nonexperts—a general practitioner, a nurse, a midwife, and a sonographer—who will receive basic training and guidance on postnatal echocardiography and probe sweeps using the SOP and instructional videos developed. Practice sessions will take place at St. Padre Pio Hospital in Douala, Cameroon. After training, the nonexperts will perform test echocardiography scans (basic sweeps) on neonates referred to the hospital to obtain video streams. Each test scan will be conducted after the baby’s routine clinical scan, with each session expected to last approximately 3 minutes, yielding a 3-minute video clip. The entire exercise is planned over 4 months, during which at least 16 neonates are expected to be scanned, producing 16 video clips. It is important to emphasize that scans performed by nonexperts are for study purposes only and will not replace the baby’s routine specialist scan. However, if deemed relevant after evaluation, the images or video clips produced by the AI model could be used by the expert to support diagnosis. For each baby, the expert’s scan will be retained alongside the test videos from the nonexperts. All videos will be deidentified using our anonymization script. Parents will receive orientation about the exercise and provide signed informed consent, with the option to decline participation (see Multimedia Appendix 3 for the information sheet and consent form).

#### Extraction and Labeling of Images/Video Clips From the Ultrasound Videos

Videos from both expert and nonexpert ultrasound sessions will be loaded into the AI model platform on the computer, allowing cardiac views (short video clips) to be extracted and sent for expert review.

#### Evaluate Extracted Images/Video Clips

The video clips will be randomly distributed to our panel of specialist pediatric cardiologists for independent evaluation. They will assess the quality of the extracted clips/frames, the correctness of the AI-assigned frame labels, and the usability of the video clips in real clinical practice, using a 5-point Likert scale. Additionally, the experts will examine the images for any CHD diagnosis as they would in routine clinical practice. At the end of the exercise, the results will be tallied, and the panel will reconvene—chaired by a designated member—to deliberate on cases with disagreements. A comparison will also be made between video clips obtained by the experts and those obtained by the nonexperts. The review process is expected to last approximately 3 months.

### Ethical Considerations and Data Security

#### Human Participant Ethics Review Approvals

Ethical approval for this study has been obtained in both Cameroon and South Africa. In Cameroon, approval was granted by the Health Research Foundation Institutional Review Board (HIRB02643525), with subsequent administrative approval obtained from the Regional Delegation of Public Health (approval number 0616/AP/MINSANTE/DRSPL/BCASS). In South Africa, ethical approval was obtained from the Human Research Ethics Committee of the University of Cape Town (UCT HREC: 697_2024). A waiver of informed consent was granted, as this study is registered as a substudy of the UCT HREC–approved PROTEA CHD registry and biorepository (UCT HREC: R017-2014). The PROTEA registry involves the collection and storage of echocardiograms, for which all participants have already provided informed consent.

In developing this methodology, we have placed particular emphasis on ethical considerations to ensure that the AI model is not only effective and efficient but also equitable, transparent, and respectful of patient rights and cultural contexts. Our approach seeks to balance technological innovation with patient safety, data privacy, and equitable distribution of benefits. Accordingly, key ethical aspects have been addressed to ensure compliance with both international standards and local regulations.

#### Informed Consent

Given the focus on neonatal research, informed consent has been made a cornerstone of the study. Parents or legal guardians of participating neonates will be fully informed about the study’s objectives, procedures, and potential benefits and risks. This ensures that participation is entirely voluntary and that parents understand how their child’s data will be used. Consent forms will be provided in appropriate local languages, and researchers will be available to answer any questions, addressing potential literacy barriers and fostering trust.

#### Privacy and Confidentiality

Neonatal echocardiography involves sensitive medical data, making confidentiality essential. All imaging data and associated patient information will be anonymized before storage or analysis. Encryption protocols and secure data transfer methods will be employed to prevent unauthorized access. These measures comply with international guidelines, including the General Data Protection Regulation, as well as the national data protection laws of the study countries.

#### Equity and Bias Mitigation

We recognize that equitable access to the benefits of the AI model is a critical ethical priority. SSA faces significant disparities in health care access, and introducing this model could inadvertently exacerbate existing inequalities if not implemented thoughtfully. By design, this study will prioritize deployment of the AI tool in low-resource settings where access to expert cardiologists is limited, ensuring that the intervention aligns with its goal of addressing health care inequities.

Another major ethical concern is the potential for bias in AI algorithms. The model’s training data must represent the diverse populations it aims to serve to ensure diagnostic accuracy across different demographic groups. Inadequate representation could lead to disparities in model performance, undermining trust, and fairness. To mitigate this, the study will incorporate data from diverse neonatal populations in Cameroon and South Africa, with plans to expand the dataset to include additional low-resource settings.

#### Transparency and Accountability

We are committed to transparency and accountability, which are integral to the ethical deployment of AI tools. Health care providers using the AI tool must understand its capabilities and limitations. The AI tool is intended to support, not replace, clinical judgment, serving as an aid for nonexperts. We will provide clear communication regarding the tool’s accuracy, error rates, and scope of applicability to prevent over-reliance and promote responsible use. Additionally, the study will establish mechanisms for continuous monitoring and reporting of any adverse events or unexpected outcomes during the implementation phase.

#### Equity, Sustainability, and Local Capacity Building

Lastly, we recognize that the long-term sustainability and scalability of the AI model may raise ethical questions regarding resource allocation and stakeholder involvement. To address this, we aim to engage local health care professionals, policy makers, and community leaders early in the process, ensuring that the model is culturally appropriate and responsive to the specific needs of the target population. Measures have been incorporated to build local capacity, training nonexperts in the use and maintenance of the AI tool. This approach reduces dependency on external entities and fosters self-reliance within the health care system.

#### Compensation Details

No financial compensation will be provided to participants. Participation is entirely voluntary, and all procedures are noninvasive and part of standard care, ensuring that families are not subjected to any additional burden beyond routine clinical interactions.

#### Image Use and Identifiability

All images used in the study will be fully anonymized, with no identifiable patient features included. If any image inadvertently poses a risk of identifiability, explicit written consent will be obtained from the parent or legal guardian, and the corresponding documentation will be submitted in accordance with ethical policies.

## Results

Retrospective data collection for the project began in September 2024. Since then, data from 308 babies have been collected, of which 145 babies’ data from Cameroon have already been labeled, while data from 163 babies in South Africa are still being transmitted. In parallel, the project model is under development, with the labeled data from Cameroon being used to initiate training. Although the initial test results are not optimal, these preliminary tests serve as a calibration step, given that all project data must be labeled and consolidated into a single dataset. Overall, the data collection process remains ongoing, and we anticipate that full data collection will be completed by October 2025. Model completion, validation, and publication of the full results will follow thereafter.

## Discussion

### Anticipated Findings

Modern advancements in data science and digital health technologies present unique opportunities to transform health care delivery in low-resource settings. This protocol outlines a structured approach for developing an AI-assisted echocardiography model designed to empower nonexperts to perform neonatal cardiac scans and capture accurate images for expert review. By addressing critical gaps in CHD diagnosis, the project represents an important step toward improving early detection and referral pathways, ultimately enhancing survival rates for neonates in SSA.

A key next step involves integrating the AI model with existing pulse oximetry and clinical ultrasound systems, alongside establishing a telemedicine platform to enable the transmission of images to remote pediatric cardiologists. One potential platform for such integration is the Commonwealth Award-winning Global Birth Defects app [20], which is designed to support accurate diagnosis of CAs for research and surveillance programs operating in low-resource settings. The app already supports the collection of data on CAs, including images and videos, which can be sent to an expert panel for evaluation. Currently, it is primarily designed for externally visible CAs. Integrating assistance with pulse oximetry and echocardiography via the CHD AI model would make the app more comprehensive, potentially transforming it from a surveillance and research tool into a clinical decision-support platform. This expansion also aligns with the mission of our new sub-Saharan African Congenital Anomaly Network [21], which could lead efforts for large-scale adoption across SSA and other LMICs.

### Comparison With Prior Work

The adoption of AI in clinical practice is not without challenges, including skepticism, ethical concerns, and regulatory requirements. To address these, the protocol incorporates processes for rigorous clinical validation and stakeholder engagement. This includes piloting the AI model with target users, assessing the quality of AI-extracted images by expert pediatric cardiologists, and collecting feedback from health care providers on the feasibility of integrating AI into routine clinical workflows. These steps ensure alignment with ethical standards and enhance the model’s clinical relevance and acceptance. As the current AI model is limited to image capture and does not provide CHD diagnoses, it is expected to foster greater acceptance among health care providers and the public.

### Strengths and Limitations

The inclusion of diverse patient populations from Cameroon and South Africa further strengthens the external validity of the proposed model. This diversity better captures regional differences in health care infrastructure and patient demographics, improving its broader applicability across SSA. Nevertheless, generalizing AI models to varied contexts remains a significant challenge [22]. Expanding the dataset to include additional SSA countries and other low-resource settings will enhance model robustness and support wider adoption.

The protocol’s imaging sequences and cardiac views adhere to the 2024 American Society of Echocardiography guidelines [16], ensuring a standardized approach to data collection. Additionally, the dual-phase model development strategy, leveraging both retrospective and prospective datasets, addresses limitations in data availability while maintaining compliance with ethical guidelines. The incorporation of metadata-driven insights and quality assurance protocols further strengthens the model’s diagnostic accuracy and clinical utility.

### Future Directions

Future updates to the current model could provide real-time feedback to guide users in probe maneuvers, enhancing both image accuracy and user expertise. As the model evolves, its scope could expand to include older pediatric populations, prenatal echocardiography, automated CHD subtype detection, and predictive analytics for treatment planning and survival outcomes. These advancements have the potential to increase the robustness and utility of the AI tool, broadening its impact on CHD management in resource-limited settings.

### Sustainability and Capacity Building

To promote long-term sustainability, the project incorporates mechanisms for real-world performance monitoring, periodic model retraining with new data, and local capacity-building through partnerships with clinical and academic stakeholders. Open-source training materials and documentation will support local ownership and reduce reliance on external experts. Recognizing the infrastructural and human factors that influence the success of mobile health (mHealth) tools in SSA—such as connectivity limitations, digital literacy gaps, and trust in digital systems [23-25]—the project emphasizes human-centered design, transparent AI behavior, and integration into existing health care structures to facilitate scalable and equitable deployment.

### Dissemination Plan

Study findings and tools will be disseminated through integration with established platforms such as the Global Birth Defects app and the sub-Saharan African Congenital Anomaly Network, promoting adoption across LMICs. Engagement with Ministries of Health, research communities, and global health bodies will support regional uptake, feedback loops, and sustainable scale-up.

### Conclusions

This protocol exemplifies a rigorous, ethical, and scalable approach to leveraging AI for neonatal echocardiography in low-resource settings. By integrating the AI model with established clinical and research standards, this unique approach will set the stage for broader development and adoption of AI technologies to improve neonatal health outcomes in SSA and other low-resource settings.

## Appendix

Multimedia Appendix 1 Study information sheet and consent form (data collection).

Multimedia Appendix 2 Statement of purpose for acquiring targeted cardiac views and video capture during echocardiographic examinations.

Multimedia Appendix 3 Study information sheet and consent form (artificial intelligence model pilot test).

Multimedia Appendix 4 Peer review report from the ZRG1 BTC-L (70) - Center for Scientific Review Special Emphasis Panel, RFA RM 22-022: DS-I Africa RFAs (National Institutes of Health, USA).

## Abbreviations

AI: artificial intelligence
CA: congenital anomaly
CHD: congenital heart disease
DS-I Africa: Data Science for Health Discovery and Innovation in Africa
HIC: high-income country
LMIC: low- and middle-income country
mHealth: mobile health
PC: prediction confidence
SOP: standard operating procedure
SSA: sub-Saharan Africa
UCT: University of Cape Town

## Data Availability

This manuscript describes a research protocol. As the study is ongoing, no datasets have yet been generated or analyzed. Upon completion of data collection and model development, anonymized echocardiographic video data and associated metadata may be made available to qualified researchers upon reasonable request, subject to ethical approvals and data-sharing agreements, in compliance with national regulations and institutional policies governing human participants’ data. Because of the sensitive nature of neonatal health data and the inclusion of video imaging, data will not be deposited in a public repository. Interested researchers may contact the corresponding author (AZL) to initiate a data access request, which will be reviewed in collaboration with the relevant ethics committees and partner institutions in Cameroon and South Africa. In the future, derived outputs such as pretrained model weights and code for preprocessing and model training will be shared via a public GitHub repository or similar open-access platform, contingent upon successful validation and compliance with institutional guidelines.
