# Supplementary material for: Artificial Intelligence–Assisted Image Extraction in Neonatal Echocardiography for Congenital Heart Disease Diagnosis in Sub-Saharan Africa: Protocol for Model Development
Source: JMIR Res Protoc. 2025 Oct 30;14:e75270. doi: 10.2196/75270 (PMC12616185; doi:10.2196/75270)
Supplement: Multimedia Appendix 1 [file resprot_v14i1e75270_app1.docx]

**Appendix A**

**Artificial Intelligence assisted echocardiography to facilitate accurate image capture and transmission for congenital heart defects diagnosis in Sub-Saharan Africa**

**Study information Sheet (Data collection)**

Dear Parent/Guardian,

We would like to invite your child to take part in an important research study. This study aims to help babies born with heart problems get the right care, even in areas where expert heart doctors are not easily available.

**Why is this study important?**

In many parts of Africa, there are very few expert children's heart doctors. This means many babies born with heart problems may not get diagnosed or treated on time. We want to find a solution to this problem.

**What is the purpose of this study?**

We are working to create a smart computer program that can identify and extract the key pictures of the heart from an ultrasound scan. This program will allow nurses, midwives and local doctors to do the scan and send the pictures to an expert heart doctor in the city. The expert can then check the pictures and make the right diagnosis. This technology can help more babies with heart problems get diagnosed and treated early.

**Why is your child being invited?**

Your child is being invited because they are scheduled for a routine heart ultrasound scan at the hospital. Taking part in this study is completely voluntary.

**What will happen if you agree?**

If you agree for your child to take part, here is what will happen during their ultrasound scan:

The technician will connect a device to record the entire scan from start to finish.

For some important heart pictures, the technician will pause and label that picture on the screen.

The full scan will be the same as usual, but may take a little longer to capture all the data needed.

The recorded video and labelled pictures will be safely sent to our research team. All personal information about your child will be kept private and removed before using the data for the study. This data can also be used for future research to improve care for babies.

**Are there any risks?**

There are no extra risks to your child beyond the routine ultrasound scan they were already scheduled for.

**What are the benefits?**

This study may lead to better access to heart care and earlier detection of heart problems for babies in your community and across the world.

**Can you withdraw?**

You have the full right to withdraw your child from this study at any time, and it will not affect their medical care.

**Will your child's information be kept private?**

Yes, all information collected will be kept strictly confidential. Your child's personal details will be removed, and the data will be stored securely.

**Who is leading this study?**

This study is led by the Health Research Foundation, Cameroon, in partnership with universities in South Africa and the UK. It is funded by the United States National Institutes of Health.

**Contact Information**

If you have any questions or concerns about this study, please contact:

- The principal investigator from HRF Cameroon: Dr. Aminkeng Zawuo Leke,email: [leke@hrfbuea.org](mailto:leke@hrfbuea.org) , Tel:+237675817024

OR

- Co-investigator from UCT SA: DR. Thomas Aldersley, email:[thomas.aldersley@uct.ac.za](mailto:thomas.aldersley@uct.ac.za)

Thank you for considering participation in this important research project

**Consent Form (Data collection)**

Consent for participation in the project: **Artificial Intelligence assisted echocardiography to facilitate accurate image capture and transmission for congenital heart defects diagnosis in Sub-Saharan Africa**

**To be completed by data collector:**

Patient Name: _________________________________________

Date: __________________________________

Name of data collector: _____________________________________

Institution/Hospital name: __________________________________________

**To be completed by person with parental responsibility:**

I have been given a Patient Information Leaflet about this project on the AI assisted echocardiography scan and an opportunity to have it explained to me and ask questions.

I understand that my baby’s echocardiography scan will be recorded and the video clip which does not contain any personal information will be used for research purposes, including the current and future research.

I understand that participation in this project will NOT influence or affect my child’s care in any way.

I understand that I may refuse to give my consent or withdraw my consent at any point and this will NOT affect the medical care given to my child in any way.

**I consent for my baby to participate in the AI assisted echocardiography project**

**Parent/Guardian**

Signature: ___________________________ Print Name: _______________________________________

Date: ________________ Relationship to child: ­­­­­­­­­­­______________________

**Data collector/Project representative**

Signature: ____________________________ Print Name: _______________________________________

Date: _________________

(For parents who cannot read or write, please confirm that you have witnessed the reading of the Parent Information Leaflet and this consent form to the parents of the child and are satisfied that they have given appropriate informed consent)

**Witness**

signature: ­­­­­­­­­­­______________________________ Print Name: ______________________________________

Date: _________________
