# Supplementary material for: Artificial Intelligence–Assisted Image Extraction in Neonatal Echocardiography for Congenital Heart Disease Diagnosis in Sub-Saharan Africa: Protocol for Model Development
Source: JMIR Res Protoc. 2025 Oct 30;14:e75270. doi: 10.2196/75270 (PMC12616185; doi:10.2196/75270)
Supplement: Multimedia Appendix 2 [file resprot_v14i1e75270_app2.docx]

**Appendix B**

**Artificial Intelligence assisted echocardiography to facilitate accurate image capture and transmission for congenital heart defects diagnosis in Sub-Saharan Africa**

**SOP for acquiring targeted cardiac views and video capture during echocardiographic examination**

| **Project: Artificial Intelligence assisted echocardiography to facilitate optimal image extraction for congenital heart defects diagnosis in Sub-Saharan Africa** | | |
| --- | --- | --- |
|  | SOP: | Echocardiogram and screen recording. |
|  | Version | 1.0 |
|  | Date | 02.2024 |
|  | Author | Thomas Aldersley, John Lawrenson |
|  | Supersedes | N/A |
|  | SOP History | N/A |
|  | Approved by |  |
|  | Approval date |  |

# **Purpose**

To describe the standard operating procedure for echocardiogram acquisition, including live screen recording.

# **Scope**

All sites performing paediatric echocardiograms for the “Artificial Intelligence assisted echocardiography to facilitate optimal image extraction for congenital heart defects diagnosis in Sub-Saharan Africa” project.

# **Procedure**

## **Setup**

1. When the patient enters the echocardiography laboratory, they will be instructed to lie down on the bed flat on their backs. Patients under two years of age will not use a pillow, Rotation onto the left lateral decubitus position will be used where necessary,
2. ECG leads will be attached for every study unless the echocardiographer is concerned that adding leads will disturb a sleeping patient.
3. All echo loops will be 4 beats long if the ECG is used or 8 seconds if the ECG is not used.
4. Patients will be sedated where necessary; caregivers will be encouraged to distract the child whenever possible.
5. Ensure that the screen recording device is appropriately attached to the echocardiogram machine. Press the record button to start the screen capture.
6. The echocardiographer will enter the patient’s details into the echo machine and begin the study.
7. Switching transducers during the study may be needed.

## **Echocardiogram**

The study should be done in the following sequence unless the patient is sedated or completely calm:

Subcostal – apex up – situs view only (IVC/AO (Can omit if patient is restless/anxious)

Apical views – apex down

Parasternal views – apex up

High-parasternal / Suprasternal – apex up

Subcostal views – apex down

1. Apical four chamber,
2. Apical 5 chamber,
3. long axis parasternal,
4. short axis parasternal,
5. parasternal short axis at level of mitral valve)
6. suprasternal short axis (for bifurcation of PA),
7. suprasternal long axis (ductal or hockey-stick arch view)

### Subcostal Views

The echocardiogram display should be orientated – **Apex-up**.

1. In the short axis – determine relationship of IVC and Aorta.
2. Rotate 90 degrees – look at pulsatility of aorta and coeliac axis, drainage of IVC into Atrium.

### Apical Views

The first cardiac views that will be obtained are the apical 4 chamber views long-axis views. The echocardiogram display should be orientated – **Apex-down.**

Measurements can be obtained during the study or from stored loops or stills after the study.

#### Apical 4 Chamber

**Label the images Apical 4 chamber**

The probe is placed on the chest where the apical beat can be felt with the marker pointing to the patient’s left.

- 1. 2D images should be stored showing the left ventricle, left atrium, right ventricle and right atrium with the mitral and tricuspid valves.
  2. The left atrium and right atrium should be traced in diastole just before the valves open.
  3. The pulmonary vein inflows can be interrogated using colour Doppler by moving the colour box distally.
  4. Colour Doppler should be placed across the mitral and tricuspid valves respectively.
  5. Continuous wave Doppler should also be taken across both of the valves if MR or TR is noted and peak velocities measured.
  6. Pulsed wave Doppler should be taken just above the mitral valve leaflet tips in the LV.
  7. Tissue Doppler should be used to measure mitral annulus velocities. . The E’ should be measured both at septal and lateral aspects of annulus.
  8. M-mode should be used at the tricuspid valve annulus to measure annular plane systolic excursion (TAPSE).
  9. The left ventricle ejection fraction must be calculated using the Simpsons biplane method. The LV cavity is traced in diastole and again in systole. This should only be done if the endocardium is clearly visible and papillary muscles must be ignored when tracing. This will necessitate turning the transducer anticlockwise to obtain the 2 Chamber view. If Global longitudinal strain is to be measured using speckle tracking the appropriate apical two chamber view must be obtain using further anticlockwise rotation.
  10. The transducer must then be turned to obtain the apical 4 chamber view again. The probe must be shifted laterally to obtain the best views of the right ventricle. The right ventricle must be measured at the basal and mid-levels during diastole. The mitral and tricuspid valve annuli should be measured and the ratio between the two calculated. The length from the RV apex to the middle of annulus must also be measured both in diastole and systole. The RV area should be traced in diastole and systole. The RV end diastolic and end systolic volume can then be calculated to determine the fractional area change.
  11. Speckle tracking can be used to measure RV free Wall strain at this time.

#### Apical 5 Chamber

**Label the images Apical 5 chamber**

The probe should then be tilted down slightly in order to also show the aortic valve.

1. Colour Doppler and continuous wave Doppler should be taken across the valve.
2. Pulsed wave Doppler must be taken in the left ventricular outflow tract just below the valve.
3. If taken at the right place, the closing click should be visible on the trace. It should then be traced to determine the LVOT VTi.
4. Further angulation may show the RV outflow and pulmonary valve – if seen measurements repeated as for aorta.

#### Apical 3 Chamber

The probe should be rotated anticlockwise until the LA and LVOT can be seen.

1. Standard interrogation of the mitral valve, LVOT and aortic valve are performed.
2. Loops of LV recorded in addition if needed to measure Global Longitudinal Strain (GLS) by speckle tracking.
3. Apical or long axis 3 Chamber is not a standard view in a routine study in our unit.

### Parasternal Long Axis

**Label the images parasternal long axis.**

The echocardiogram display should be orientated – **Apex-up**.

The probe is placed on the patients’ chest on the left side at about the third intercostal space and rotated so that the marker is pointing to the right shoulder.

1. A 2D image showing the left atrium (LA), left ventricle (LV), mitral and aortic (Ao) valves, RV, and ascending Ao, should be obtained.
2. M-mode measurements are taken across the aorta and left atrium, measuring the aortic root and left atrium from inner edge to inner edge.
3. M-mode measurement should also be taken across the left ventricle at the level of the papillary muscles. Dimensions, including the interventricular septum, left ventricular internal diameter and posterior wall thickness, should be measured both in diastole and systole.
4. Colour Doppler interrogation of mitral valve and aortic valve should be performed.
5. Slight movement of the probe to the patient’s left will facilitate measurement of aortic annulus, sinus and sinotubular junction measurements.
6. Rightward and inferior angulation toward right hip shows right atrium (RA), tricuspid valve, and right ventricular (RV) inflow. Coronary sinus can be followed into RA in this view.
7. In this view, colour Doppler interrogation of the tricuspid valve inflow should be performed; TR velocity should be measured using CW.
8. Moving the probe slightly up the chest and leftward with angulation towards the left shoulder will show the main PA and LPA. Colour Doppler should be used to look for a duct.

### Parasternal Short Axis

**Label view parasternal short axis views.**

The echocardiogram display should be orientated – **Apex-up**.

The probe should then be rotated till the marker points at the left shoulder in order to obtain the parasternal short-axis view.

1. A 2D image showing the aortic cusps, pulmonary, mitral, and tricuspid valves should be obtained.
2. The R and L coronary arteries should be visualised.
3. Colour Doppler should be placed across all three valves respectively. The probe should be tilted up slightly and colour Doppler should then be placed across the pulmonary artery showing the bifurcation.
4. Continuous wave Doppler should be taken across the pulmonary and tricuspid valves. If there is tricuspid regurgitation present, the peak velocity will be measured.
5. The probe should then be tilted down and 2D images should be acquired at the level of the mitral valve, papillary muscles and the LV apex in the parasternal short-axis view.

### High-parasternal/Suprasternal views

**Label view as Suprasternal views**

The echocardiogram display should be orientated – **Apex-up**.

The probe must be moved higher up the chest - with the indicator in the 12 o’clock position.

1. The aortic arch and descending aorta as well as the head and neck vessels must be evaluated. CW and colour Doppler must be used to evaluated flow in the arch.
2. The diameters of the proximal and distal arch and isthmus must be measured.

The probe should be rotated to the three o’clock position

1. In this view the branches of the aorta can be evaluated as can diameter of the RPA.
2. In small patients the pulmonary veins can be seen entering the LA – used colour Doppler to demonstrate the flows.

### Subcostal views

**Label images as Subcostal Views**

The echocardiogram display should be orientated – **Apex-down**.

The probe should then be placed at the subcostal position with the marker pointing to the patient’s left (3 o’clock).

1. A 2D image showing all four chambers should be stored.
2. Colour Doppler can be placed across the intra-atrial septum.
3. The probe should then be rotated to point to the patient’s feet
4. The inferior vena cava and superior vena can then be assessed
5. Rotating to the 5 o'clock position will allow assessment of the atrial septum, Tilting the probe towards the abdomen will show the LV outflow tract
6. Rotating to 2 o'clock will show the RV outflow tract

This echocardiogram SOP is based on the 2024 American Society for Echocardiography guidelines. DOI: [https://doi.org/10.1016/j.echo.2023.11.015](https://protect.checkpoint.com/v2/r02/___https://doi.org/10.1016/j.echo.2023.11.015___.YzJlOnVsc3RlcnVuaXZlcnNpdHk6YzpvOjdiMjU0OTk1MDZmYTQ0MGZhMjQwNzc5ZjEzYzEyNTcxOjc6ZWNmYjo5NjkyYTliYjAwYTJkOTVkNTlkODZmMTNhY2IyM2U5ZjgzNWRkMTFhOTY0NDdkZTk1YWIyZDJhMzMzOTUzNzBkOnA6VDpO)
