# Supplementary material for: Artificial Intelligence–Assisted Image Extraction in Neonatal Echocardiography for Congenital Heart Disease Diagnosis in Sub-Saharan Africa: Protocol for Model Development
Source: JMIR Res Protoc. 2025 Oct 30;14:e75270. doi: 10.2196/75270 (PMC12616185; doi:10.2196/75270)
Supplement: Multimedia Appendix 3 [file resprot_v14i1e75270_app3.docx]

**Appendix C**

**Artificial Intelligence assisted echocardiography to facilitate accurate image capture and transmission for congenital heart defects diagnosis in Sub-Saharan Africa**

**Study information Sheet (AI model pilot test)**

**Introduction**

You are invited to participate in a research study that aims to address the problem of congenital heart defect diagnosis in low resource settings such as sub-Saharan Africa (SSA). As you may know, the diagnose congenital heart defects in neonates requires and echocardiography scan and interpretation of the results by an expert paediatric cardiologist. Unfortunately, there are very limited expert cardiologists in SSA, most of whom are located only in large urban hospitals in the major cities. This means most babies born in rural communities may not have access to these services or have to travel long distances to reach the city. Ultimately, these babies may end up not being diagnosed, or diagnosed or receive care late or may die. A solution is urgently needed to address this problem.

**Purpose of the Study:**

The purpose of this study is to test an AI model that has been developed by our project team to help non expert cardiologist such as nurses, midwives, sonographers, and doctors to neonatal echocardiography scan and accurately identify and extract the key cardiac views. The images extracted from the scan can be subsequently sent to a remote expert cardiologist in the city for interpretation and diagnosis. This technology has the potential to enhance the diagnosis of congenital heart defects, improve access to care and increase overall survival of babies born with congenital heart defects in SSA and other low resource settings.

**Participant Selection**

You are being invited to participate in this study because your child is scheduled to undergo a routine echocardiography examination at the hospital. Your child's participation in this study is completely voluntary.

**Procedures**

If you agree to participate, the following will happen after your child's primary echocardiography scan:

- A trained non-expert cardiologist (e.g. nurses, midwives, doctor, sonographer) will conduct a quick test scan (about 5 mins) of your baby’s heart
- This test scan will be conducted only after the expert cardiologist must have completed your baby’s echocardiophy scan
- Prior to the test scan, a screen capture device will be connected to the ultrasound machine to record the entire scan from start to finish.

The recorded video will be securely transmitted to the research team for analysis. Your child's personal information will be kept confidential, and the data will be anonymized before being used for the study. The data will be stored and can be used for other future research to improve care of babies.

**Risks and Benefits**

There are no additional risks to your child beyond the routine echocardiography examination. The potential benefits of this study include improved access to cardiac care and earlier detection of congenital heart defects in your community.

**Voluntary Participation and Withdrawal**

Your child's participation in this study is completely voluntary. You have the right to withdraw your child from the study at any time without affecting their medical care.

**Confidentiality**

All information collected during this study will be kept strictly confidential. Your child's personal information will be anonymized, and the data will be stored securely on a password-protected platform.

**Project Institutions**

This study is lead by Health Research Foundation, Cameroon, in collaboration with the University of Cape Town-SA, Ulster University-UK, Kings College London and Imperial College London. The project is Funded by the United States National Institute of Health.

**Contact Information**

If you have any questions or concerns about this study, please contact:

- The principal investigator from HRF Cameroon: Dr. Aminkeng Zawuo Leke, email:[leke@hrfbuea.org](mailto:leke@hrfbuea.org) , Tel:+237675817024

Thank you for considering participation in this important research project

**Consent Form (AI model pilot test)**

Consent for participation in the project: **Artificial Intelligence assisted echocardiography to facilitate accurate image capture and transmission for congenital heart defects diagnosis in Sub-Saharan Africa**

**To be completed by data collector:**

Patient Name: _________________________________________

Date: __________________________________

Name of data collector: _____________________________________

Institution/Hospital name: __________________________________________

**To be completed by person with parental responsibility:**

I have been given a Patient Information Leaflet about this project on the AI assisted echocardiography scan and an opportunity to have it explained to me and ask questions.

I understand that after my baby’s primary echocardiography scan by an expert cardiologist, a test echocardiographer scan will be conducted by a trained non-expert cardiologist (e.g. nurse, midwife, sonographer, doctors)

I understand the test scan will be recorded and the video clip which does not contain any personal information will be used for research purposes, including the current and future research.

I understand that participation in this project will NOT influence or affect my child’s care in any way.

I understand that I may refuse to give my consent or withdraw my consent at any point and this will NOT affect the medical care given to my child in any way.

**I consent for my baby to participate in the AI assisted echocardiography project**

**Parent/Guardian**

Signature: ___________________________ Print Name: _______________________________________

Date: ________________ Relationship to child: ­­­­­­­­­­­______________________

**Data collector/Project representative**

Signature: ____________________________ Print Name: _______________________________________

Date: _________________

(For parents who cannot read or write, please confirm that you have witnessed the reading of the Parent Information Leaflet and this consent form to the parents of the child and are satisfied that they have given appropriate informed consent)

**Witness**

signature: ­­­­­­­­­­­______________________________ Print Name: ______________________________________

Date: _________________
