# Supplementary material for: Artificial Intelligence–Assisted Image Extraction in Neonatal Echocardiography for Congenital Heart Disease Diagnosis in Sub-Saharan Africa: Protocol for Model Development
Source: JMIR Res Protoc. 2025 Oct 30;14:e75270. doi: 10.2196/75270 (PMC12616185; doi:10.2196/75270)
Supplement: Multimedia Appendix 4 [file resprot_v14i1e75270_app4.pdf]

SUMMARY STATEMENT

(Privileged Communication)

PROGRAM CONTACT:
Kristin Burns
301-594-6859
burnskr@mail.nih.gov

Release Date: 03/31/2023
Revised Date:

Application Number: 1 U01 HL172179-01
Formerly: 1U01TW012519-01

Principal Investigator

LEKE, AMINKENG ZAWUO

Applicant Organization: HEALTH RESEARCH FOUNDATION

Review Group: ZRG1 BTC-L (70)
Center for Scientific Review Special Emphasis Panel
RFA RM 22-022: DS-I Africa RFAs

Meeting Date: 03/15/2023
Council: MAY 2023
Requested Start: 09/04/2023
RFA/PA: RM22-023
PCC: HH N
Dual IC(s): RM

Project Title: Artificial Intelligence assisted echocardiography to facilitate optimal image extraction for congenital heart defects diagnosis in Sub-Saharan Africa
SRG Action: Impact Score:30
Next Steps: Visit https://grants.nih.gov/grants/next_steps.htm
Human Subjects: 30-Human subjects involved - Certified, no SRG concerns
Animal Subjects: 10-No live vertebrate animals involved for competing appl.
Gender: 4A-Gender representation unknown, scientifically acceptable
Minority: 5A-Only foreign subjects, scientifically acceptable
Age: 1A-Children, Adults, Older Adults, scientifically acceptable

Project Year	Direct Costs Requested	Estimated Total Cost
1	196,135	229,498
2	170,635	199,660
3	165,831	194,039
TOTAL	532,601	623,196

NEW INVESTIGATOR

1U01HL172179-01 LEKE, Aminkeng

NEW INVESTIGATOR

RESUME AND SUMMARY OF DISCUSSION: The investigator plans to address the problem of congenital heart defects (CHD) diagnosis in Sub-Saharan Africa (SSA) by creating the possibility for low skilled sonographers to conduct an echocardiography scan for neonates (0-28 days) and extract optimal images that can be subsequently transmitted to a remote expert for interpretation. Reviewers agree that the significance is high because it successful, there will be a new working pipeline to improve CHD diagnosis on SSA. The premise of the work may have been better supported by discussion on the current literature in the field. The research team is considered excellent with complimentary skill sets. Strengths of the approach are the training plans and the expert panel that has been recruited for the truthing. Image validation is possible. One weakness is that the project may be too ambitious for the scope if this U01 award mechanism. Some reviewers were concerned with the lack of a formal assessment of interrater reliability. Also, the model does not include the use of transformers. Overall, the strengths of the significance, approach, and investigators outweighed the weaknesses and reviewers agree that the project will have a high impact on the CHD diagnosis field.

DESCRIPTION (provided by applicant): Artificial Intelligence assisted echocardiography to facilitate optimal image extraction for congenital heart defects diagnosis in Sub-Saharan Africa Summary Sub-Saharan Africa (SSA) accounts for over 50% of all global under-5 deaths. Congenital anomalies (CAs), notably congenital heart defects (CHD) which constitutes about a third of all (CAs), are a major contributor to this high under-5 morbidity and mortality in SSA. Late and missed diagnosis, owing to the lack of experts who can perform an echocardiography scan, remains the primary challenge to CHD diagnosis and care in SSA. Recently, there have been increased uptake CHD screening in newborns by pulse oximetry. However, the test is nonspecific and still requires expert confirmation through echocardiography. The few expert paediatric cardiologist centres that exist are often located hundreds of kilometers away from the birthing centres, placing enormous financial and physical burden on parents who must undertake this journey to confirm their baby's diagnosis, and not leaving out the particularly fragile and vulnerable neonate who may end up dying in the course of the journey. Training programs have been demonstrated to improving image capture and recognition of the anomaly. However, such programs are labor and time intensive and need to be repeated with staff turnover. A complementary strategy is therefore needed to improve and sustain the gains from training. In line with the DSI-Africa's mission to address critical health gaps through the application of data science, our proposed project seeks to leverage modern advances in data science and artificial intelligence (AI) to address the problem of CHD diagnosis in SSA by creating the possibility for low skilled sonographers to conduct an echocardiography scan for neonates (0-28 days) and extract optimal images that can be subsequently transmitted to a remote expert for interpretation. This means local non-experts (e.g., GPs, nurses, midwives) serving the birthing centres will now be able conduct postnatal echocardiography scans for neonates suspected of having a CHD after pulse oximetry screening, allowing them to obtain optimal labeled images/video clips that can be transmitted to a remote expert for diagnosis confirmation. This will remove the burden and risk of travelling hundreds of kilometers, increase early diagnosis and initiation of care remotely, and reduce the workload on the few available experts. Future steps will include extending to prenatal diagnosis and predicting actual diagnosis.

PUBLIC HEALTH RELEVANCE: Artificial Intelligence assisted echocardiography to facilitate optimal image extraction for congenital heart defects diagnosis in Sub-Saharan Africa Narrative Congenital heart defects (CHD) is a major contributor to under-5 deaths in Sub-Saharan Africa (SSA), owing to the lack of paediatric cardiologist experts who can perform an echocardiography scan for early diagnosis. The current project seeks to develop an artificial intelligence assisted model that will allow local non-

experts such as nurses who work in birthing centres, to conduct an echocardiography scan and extract optimal images which can sent to a remote expert for interpretation and diagnosis confirmation. This will remove the burden and risk of travelling hundreds of kilometers to expert centres mainly located in cities, increase early diagnosis and initiation of care remotely, and reduce the workload on the few available experts.

CRITIQUE 1

Significance: 4
Investigator(s): 1
Innovation: 1
Approach: 4
Environment: 2

Overall Impact: The proposal addresses the need for improved diagnosis of congenital heart defects which is a problem contributing to higher death rates among children less than 5 years old in Sub-Saharan Africa. The proposal offers an innovative approach to this problem by providing an AI assisted tool to allow less skilled sonographers to obtain images or video clips from echocardiography and then send these to experts for diagnosis of congenital heart defects. To build this system the proposed approach has 5 objectives/steps. The research team consists of experts with the qualifications to conduct these steps. A major weakness of the approach is that each step has enough complexity that the proposal does not have sufficient evidence that it can be accomplished successfully. For example, the only evidence for the performance of the AI technique at the heart of the approach is with an unrelated problem used for comparing similar algorithms with each other but not for judging performance on the specific problem of congenital heart defect diagnosis from echocardiography. Other weaknesses include the failure to define evaluation criteria, formally assess interrater reliability, and describe the basis for their procedural SOPs. This proposal may have a high impact on research supporting diagnosis of congenital heart disease, but it does have weaknesses.

1. Significance:

Strengths

- The proposal effectively justifies the need for research into AI supported methods to enable more extensive capabilities for detecting congenital heart defects using images from echocardiography. The specific objectives in the proposal provide a clear roadmap to the development of an AI system for use by sonographers to extract selected images from echocardiography videos and then make those available for expert diagnosis. If successful, the project could provide a foundation for development of an effective approach to use prenatal echocardiography.

Weaknesses

- The number and complexity of the steps in this research means that many of the steps are not well formulated or lack sufficient evidence to justify the methods proposed. The proposal does not provide preliminary results or testing outcome for any of the methods or techniques described in the list of steps.

2. Investigator(s):

Strengths

- The principal investigator is a new investigator, but he has good experience with congenital anomaly research. His team of co-investigators provides well-qualified support for this project in areas from machine learning and computer vision to pediatric cardiology.

Weaknesses

- None noted.

3. Innovation:

Strengths

- The proposed deployment of an AI-based system for use by lower skilled sonographers to identify areas of images for video clips of significance for follow-on review represents an important innovation with good applicability to other health problems in Africa.

Weaknesses

- None noted.

4. Approach:

Strengths

- The approach has a clear set of objectives that if implemented could produce an initial system for deployment testing.
- The proposal provides a good a contingency plan with approaches for many of the possible adverse events they may face during this research. For instance, labeling is a major bottleneck for supervised learning. The time proposed for labeling seems unrealistically low. The PI has anticipated this potential problem and has included the possibility to bring in additional pediatric cardiologists to help with this task.

Weaknesses

- The approach is based on the application of the Looking Fast and Slow model. This model uses two feature extraction subnetworks that reduce computational redundancy. Since the publication of this approach in 2019 other methods, most notably using transformers have been developed and shown to produce improved accuracy at higher frame rates. The proposal team has not attempted an initial test of their approach for the echocardiography and the only results available are those provided by the approach authors who applied it to ImageNet data. This testing is not applicable to the proposed problem.
- The labeling of images is done by experts working independently and then brought together to form consensus. This is a well-studied problem that can be improved using interrater reliability measures.
- The de-identification of the videos and images is done by creating a new separate number. This is a well-known problem in privacy protection that can be better handled by hashing the patient id and using a trusted third party. This will both protect patient privacy and enable patient look-up as needed for validation and quality assurance.
- The proposal discusses developing SOPs from relevant literature but does not define this literature.

- The evaluation of extracted images/video clips from the AI system will be done by a panel of experts using undefined criteria.
- If their supervised learning approach over-fits they will use cross-validation. Cross-validation is actually a useful step to detect over-fitting but does not provide a solution to the problem.

5. Environment:

Strengths

- The facilities at the Health Research Foundation (HRF) Buea provide adequate support for the research. The Digital Technology Hub with its team of engineers will be particularly useful for this research.

Weaknesses

- None noted.

Protections for Human Subjects

Acceptable Risks and/or Adequate Protections.

- Good plan for protection of patients.

Data and Safety Monitoring Plan (Applicable for Clinical Trials Only):

Inclusion Plans

- Sex/Gender: Distribution justified scientifically.
- Race/Ethnicity: Distribution justified scientifically.
- For NIH-Defined Phase III trials, Plans for valid design and analysis:
- Inclusion/Exclusion Based on Age:

Vertebrate Animals

Not Applicable (No Vertebrate Animals).

Biohazards

Not Applicable (No Biohazards).

Applications from Foreign Organizations

Justified.

- Justified by the FOA.

Resource Sharing Plans

Acceptable.

Authentication of Key Biological and/or Chemical Resources

Not Applicable (No Relevant Resources).

Budget and Period of Support

Recommend as Requested.

CRITIQUE 2

Significance: 2
Investigator(s): 2
Innovation: 2
Approach: 2
Environment: 2

Overall Impact: This proposal addresses a very significant issue in developing machine learning / artificial intelligence (AI/ML) in detecting congenital abnormalities (CAs). Congenital heart defects (CHDs) are one such CA which is currently a major contributor to under-5 deaths in Sub-Saharan Africa (SSA) therefore the impact of the proposed work is high in Africa owing to the lack of pediatric cardiologist experts who can perform an echocardiography scan for early diagnosis. The proposed approach seeks to developing AI/ML models that will allow local non-experts such as nurses who work in birthing centers, to conduct an echocardiography scan and extract optimal images which can sent to a remote expert for interpretation and diagnosis confirmation. The project is technically innovative with a reinforcement learning algorithm that uses an adaptative interleaving policy framework based on reinforcement with convolutional Long Short-Term Memory (convLSTM) to identify the optimal frames. The strong team from Cameroon for overall coordination of a large number with experts in epidemiology, pediatric cardiology, engineering, as well as experts to fill educational and administrative roles required to manage a large cadre of supporting co-investigators from other countries including expert 'readers' of medical imaging pediatric ultrasounds. The PIs have impressively worked together to create the innovative Global Birth Defects App and to establish the sub- Saharan African Congenital Anomaly Network (sSCAN) which includes an additional network experts and stakeholders on CAs across Africa. In conclusion, the potential overall impact of this project remains high because successful completion may remove the burden and risk of travelling hundreds of kilometers to expert centers mainly located in cities, increase early diagnosis and initiation of care remotely, and reduce the workload on the few available experts thereby reducing the mortality and morbidity of disease across the spectrum of CAs.

1. Significance:

Strengths

- SSA where which accounts for over 50% of all global under-5 deaths and congenital anomalies (CAs) such as congenital heart defects (CHD) are a major contributor to this mortality and morbidity.
- Late and missed diagnosis, owing to the lack of experts who can perform an echocardiography scan, remains the primary challenge to CHD diagnosis and care in SSA.
- Pediatric cardiologists are located usually in large cities hundreds of kilometers away from the birthing centers and out of reach physically and financially to poor rural patients.

Weaknesses

- None noted.

2. Investigator(s):

Strengths

- The entire project will be led by the team from Cameroon (overall coordination and epidemiology (Dr. Leke), Paediatric cardiology (Dr. Tanchou), Engineering (Dr. Dr. Deffo and Mr. Emmanuel), educational and administrative Dr. Nkwati) with support from co-investigators from other countries.
- Diverse team with complementing skillsets including 2 epidemiologists (Dr. Leke (PI) and Prof. Dolk), 5 paediatric cardiologists (Dr. Tanchou, Prof. Casey, Dr. Day, Dr. Aldersley, prof Zühlke) and Expert readers in UK and SSA.
- Strong prior interaction between Dr. Leke has years of experience working in Cameroon and with Prof. Dolk (Ulster University, UK) where they have created the innovative Global Birth Defects App and establish the sub- Saharan African Congenital Anomaly Network (sSCAN).
- sSCAN includes experts and stakeholders on CAs across Africa, including Dr. Tanchou and Dr. Nkwati from Cameroon and Dr. Aldersley and Prof. Zühlke from SA.
- Strong and experienced team of experts to handle the computer engineering tasks of the project (Dr. Deffo, Mr. Tchokote, Prof. Kainz, Dr. McAllister Prof. King, Prof. Bond, Prof. Dewar and prof Mishra).
- Educational and administrative tasks will be led by Dr. NKwati from Cameroon who has extensive experience in similar roles, with support from Dr. Leke.

Weaknesses

- None noted.

3. Innovation:

Strengths

- Goal of the training will be for the non-expert to be able to stream a good video of the baby's heart with no expectation to capture the correct image frame/video clip as this will be done by the AI model.
- Leveraging modern advances in data science and artificial intelligence (AI) to address the problem of CHD diagnosis in SSA is innovative.
- Proposed solution will allow for low skilled sonographers to conduct an echocardiography scan for neonates (0-28 days) and extract optimal images that can be subsequently transmitted to a remote expert for interpretation.
- Facilitates local non-experts (e.g., GPs, nurses, midwives) that are in rural birthing centers will now be able conduct postnatal echocardiography scans for neonates suspected of having a CHD to obtain optimal labeled images/video clips that can be transmitted to a remote expert for diagnosis confirmation.
- This hybrid approach will leverage AI and telemedicine to remove the burden and risk of travelling hundreds of kilometers, increase early diagnosis and initiation of care remotely, and reduce the workload on the few available experts.

- Proposal is practical as future work may extend their approach to prenatal diagnosis and predicting actual diagnosis.

Weaknesses

- None noted.

4. Approach:

Strengths

- Novel approach similar to Mason et al., (2019)27 for learning an adaptative interleaving policy using reinforcement learning.
- Novel interleaved framework where two feature extractors with drastically different speeds and recognition capabilities will be run on different frames.
- Convolutional Long Short-Term Memory (convLSTM) layer and detections are generated by fusing context from previous frames with the gist from the current frame.
- Option to add a feature that will enable the model to provide real-time feedback to the user during a scan.

Weaknesses

- None noted.

5. Environment:

Strengths

- Large network of clinical sites is important for validation.

Weaknesses

- None noted.

Study Timeline:

Strengths

- Excellent.

Weaknesses

- None noted.

CRITIQUE 3

Significance: 3
Investigator(s): 2
Innovation: 4
Approach: 3
Environment: 2

Overall Impact: This is a well thought out proposal. Strengths includes allocation of sufficient effort to acquire, clean and label the training and testing datasets. Expert opinion panel with provide truthing. CNN architecture seems appropriate. Concerns would be related to the computing power needed, the short time that can be allowed for the video processing given the high frame rate of echocardiography, the data bandwidth limitations for transferring the video data, and the generalizability of the method.

1. Significance:

Strengths

- This application seeks to address a critically important problem, because sub-Saharan Africa accounts for over 50% of global under-5 deaths.
- Builds upon an existing and successful method developed by Mason et al (2019).
- There is a large amount of available data, and there is a recognition of the need for good data cleaning and truthing.

Weaknesses

- It is unclear how the video processing will ultimately work, and if sufficient computer power is available, and if it is possible to stream the data are a sufficiently high rate.

2. Investigator(s):

Strengths

- A very skilled team has been assembled, including epidemiologists, pediatric cardiologists, and computer scientists.

Weaknesses

- None noted.

3. Innovation:

Strengths

- Application to neonates in SSA.

Weaknesses

- The AI methods are fairly well established. While a strength in terms of the approach, this can weaken the innovation. Other more powerful techniques such as transformers should have at least been discussed to support the decision to follow the work of Mason.

4. Approach:

Strengths

- A large number of pre-existing images (90,100 still images, 26,500 videos) will be used. Staffing is allocated for data preparation.
- A panel of experts will perform truthing.
- The architecture, training, testing, validation, etc. are well thought out.

Weaknesses

- The method for real-time feedback presumes the forward model is sufficiently fast (typically echo runs at 40-80 fps, giving just 12-15 ms per frame). The method proposed has not been used for images at this frame rate.
- The method proposed has not been used for medical ultrasound.
- No details are given on the echocardiographic equipment that the images were acquired on, or the equipment that would be used going forward. Developing an AI system for use with arbitrary imaging equipment is generally quite difficult.

5. Environment:

Strengths

- The environment is sufficient to the task.

Weaknesses

- None noted.

Protections for Human Subjects

Acceptable Risks and/or Adequate Protections.

- The enrollment table is blank, but otherwise the study seems sound.

Data and Safety Monitoring Plan (Applicable for Clinical Trials Only):

Not Applicable (No Clinical Trials).

Inclusion Plans

- Sex/Gender: Distribution justified scientifically.
- Race/Ethnicity: Distribution justified scientifically.
- For NIH-Defined Phase III trials, Plans for valid design and analysis: Not applicable.
- Inclusion/Exclusion Based on Age: Distribution justified scientifically.
- Study restricted to neonates.

Vertebrate Animals

Not Applicable (No Vertebrate Animals).

Biohazards

Not Applicable (No Biohazards).

Applications from Foreign Organizations

Justified.

- This RFA is specifically intended for institutions based in Africa.

Resource Sharing Plans
Acceptable.

Authentication of Key Biological and/or Chemical Resources
Not Applicable (No Relevant Resources).

Budget and Period of Support
Recommend as Requested.

THE FOLLOWING SECTIONS WERE PREPARED BY THE SCIENTIFIC REVIEW OFFICER TO SUMMARIZE THE OUTCOME OF DISCUSSIONS OF THE REVIEW COMMITTEE, OR REVIEWERS' WRITTEN CRITIQUES, ON THE FOLLOWING ISSUES:

PROTECTION OF HUMAN SUBJECTS: ACCEPTABLE

INCLUSION OF WOMEN PLAN: ACCEPTABLE

INCLUSION OF MINORITIES PLAN: ACCEPTABLE

INCLUSION ACROSS THE LIFESPAN: ACCEPTABLE

COMMITTEE BUDGET RECOMMENDATIONS: The budget was recommended as requested.

Footnotes for 1 U01 HL172179-01; PI Name: LEKE, Aminkeng Zawuo

NIH has modified its policy regarding the receipt of resubmissions (amended applications).See Guide Notice NOT-OD-18-197 at https://grants.nih.gov/grants/guide/notice-files/NOT-OD-18-197.html. The impact/priority score is calculated after discussion of an application by averaging the overall scores (1-9) given by all voting reviewers on the committee and multiplying by 10. The criterion scores are submitted prior to the meeting by the individual reviewers assigned to an application, and are not discussed specifically at the review meeting or calculated into the overall impact score. Some applications also receive a percentile ranking. For details on the review process, see http://grants.nih.gov/grants/peer_review_process.htm#scoring.

MEETING ROSTER

Center for Scientific Review Special Emphasis Panel
CENTER FOR SCIENTIFIC REVIEW
RFA RM 22-022: DS-I Africa RFAs

ZRG1 BTC-L (70)
03/15/2023 - 03/16/2023

Notice of NIH Policy to All Applicants: Meeting rosters are provided for information purposes only. Applicant investigators and institutional officials must not communicate directly with study section members about an application before or after the review. Failure to observe this policy will create a serious breach of integrity in the peer review process, and may lead to actions outlined in NOT-OD-22-044 at https://grants.nih.gov/grants/guide/notice-files/NOT-OD-22-044.html, including removal of the application from immediate review.

CHAIRPERSON(S)

WHALEN, CHRISTOPHER C, MD
PROFESSOR
DEPARTMENT OF EPIDEMIOLOGY AND BIOSTATISTICS
COLLEGE OF PUBLIC HEALTH
UNIVERSITY OF GEORGIA
ATHENS, GA 30602

MEMBERS

ADEBAMOWO, SALLY NNEOMA, MBBS, SCD
ASSOCIATE PROFESSOR
DEPARTMENT OF EPIDEMIOLOGY AND PUBLIC HEALTH
SCHOOL OF MEDICINE
UNIVERSITY OF MARYLAND
BALTIMORE, MD 21201

ALEKSEYENKO, ALEXANDER V, BS, MS, PHD, POSTDOC
PROFESSOR
BIOMEDICAL INFORMATICS CENTER
DEPARTMENT OF PUBLIC HEALTH SCIENCES
MEDICAL UNIVERSITY OF SOUTH CAROLINA
CHARLESTON, SC 29425

BLOWER, SALLY MARGARET, PHD
PROFESSOR
DEPARTMENT OF PSYCHIATRY
AND BIOBEHAVIORAL SCIENCES
DAVID GEFFEN SCHOOL OF MEDICINE
UNIVERSITY OF CALIFORNIA, LOS ANGELES
LOS ANGELES, CA 90024

BOLLINGER, ROBERT C JR, MD, MPH
PROFESSOR
CENTER FOR CLINICAL GLOBAL HEALTH EDUCATION
THE JOHNS HOPKINS UNIVERSITY SCHOOL OF MEDICINE
BALTIMORE, MD 21287

BRAUN, TERRY A, PHD
PROFESSOR
DEPARTMENT OF BIOMEDICAL ENGINEERING
COLLEGE OF ENGINEERING
UNIVERSITY OF IOWA
IOWA CITY, IA 52242

BROWN, DONALD E, PHD
SENIOR ASSOCIATE DEAN
QUANTITATIVE FOUNDATION DISTINGUISHED
PROFESSOR OF DATA SCIENCE
SCHOOL OF DATA SCIENCE
UNIVERSITY OF VIRGINIA
CHARLOTTESVILLE, VA 22904

BUCHER, SHERRI LYNN, PHD
ASSOCIATE PROFESSOR
DEPARTMENT OF PEDIATRICS
SCHOOL OF MEDICINE
INDIANA UNIVERSITY
INDIANAPOLIS, IN 46202

EZEH, ALEX, PHD
PROFESSOR
DEPARTMENT OF COMMUNITY HEALTH AND PREVENTION
DORNSIFE SCHOOL OF PUBLIC HEALTH
DREXEL UNIVERSITY
PHILADELPHIA, PA 19104

GIGNOUX, CHRISTOPHER R, PHD
ASSOCIATE PROFESSOR
DIRECTOR OF RESEARCH
COLORADO CENTER FOR PERSONALIZED MEDICINE
ANSHUTZ MEDICAL CAMPUS
UNIVERSITY OF COLORADO
AURORA, CO 80045

HADLEY, DEXTER D, MD, PHD
ASSISTANT PROFESSOR
DEPARTMENT OF PATHOLOGY
UNIVERSITY OF CENTRAL FLORIDA
ORLANDO, FL 32827

HARRINGTON, LAURA C, PHD
PROFESSOR
DEPARTMENT OF ENTOMOLOGY
CORNELL UNIVERSITY
ITHACA, NY 14853

HUBBARD, ALAN E
ASSOCIATE PROFESSOR
DIVISION OF BIOSTATISTICS
SCHOOL OF PUBLIC HEALTH
UNIVERSITY OF CALIFORNIA, BERKELEY
BERKELEY, CA 94720

JENKINS, HELEN E, PHD
ASSOCIATE PROFESSOR
DEPARTMENT OF BIOSTATISTICS
SCHOOL OF PUBLIC HEALTH
BOSTON UNIVERSITY
BOSTON, MA 02118

KARIUKI, SAMUEL M, DVM, MSC, PHD, BVM
CENTER FOR MICROBIOLOGY RESEARCH
DEPARTMENT OF MICROBIOLOGY
KENYA MEDICAL RESEARCH INSTITUTE
NAIROBI 00100
KENYA

LUKE, AMY H, PHD
PROFESSOR
DEPARTMENT OF PUBLIC HEALTH SCIENCES
PARKINSON SCHOOL OF HEALTH SCIENCES
AND PUBLIC HEALTH
LOYOLA UNIVERSITY CHICAGO
MAYWOOD, IL 60153

MACHEKANO, RHODERICK, PHD, MPH, MS
BIO STATISTICIAN
SCHOOL OF MEDICINE
STELLENBOSCH UNIVERSITY
STELLENBOSCH
SOUTH AFRICA

MAIDMENT, ANDREW DOUGLAS ARNOLD, PHD
ASSOCIATE PROFESSOR
DEPARTMENT OF RADIOLOGY
PERELMAN SCHOOL OF MEDICINE
UNIVERSITY OF PENNSYLVANIA
PHILADELPHIA, PA 19104

NDEBELE, PAUL, PHD
SENIOR RESEARCH REGULATORY SPECIALIST AND
PROFESSIONAL LECTURER
DEPARTMENT OF GLOBAL HEALTH
MILKEN INSTITUTE SCHOOL OF PUBLIC HEALTH
GEORGE WASHINGTON UNIVERSITY
WASHINGTON, DC 20052

PUYANA, JUAN CARLOS, MD
PROFESSOR
DEPARTMENT OF SURGERY, CRITICAL CARE MEDICINE,
AND CLINICAL TRANSLATIONAL SCIENCE
SCHOOL OF MEDICINE
UNIVERSITY OF PITTSBURGH
PITTSBURGH, PA 15213

ROSENBERG, NORA, PHD
ASSOCIATE PROFESSOR
DEPARTMENT OF HEALTH BEHAVIOR
GILLINGS SCHOOL OF GLOBAL HEALTH
UNIVERSITY OF NORTH CAROLINA
CHAPEL HILL, NC 27599

SALIHU, HAMISU, MD, MBBS, PHD
PROFESSOR AND VICE CHAIR
BCM CENTER OF EXCELLENCE
IN HEALTH EQUITY, TRAINING & RESEARCH
BAYLOR COLLEGE OF MEDICINE
HOUSTON, TX 77098

SHARP, ANDREW JAMES, PHD
ASSOCIATE PROFESSOR
DEPARTMENT OF GENETICS AND
GENOMIC SCIENCES
ICAHN SCHOOL OF MEDICINE AT MOUNT SINAI
NEW YORK, NY 10029

STINGONE, JEANETTE A, MPH, PHD
ASSISTANT PROFESSOR
DEPARTMENT OF EPIDEMIOLOGY
MAILMAN SCHOOL OF PUBLIC HEALTH
COLUMBIA UNIVERSITY
NEW YORK, NY 10032

TURNER, ELIZABETH L, PHD
ASSOCIATE PROFESSOR
DIRECTOR, DGHI RESEARCH DESIGN AND ANALYSIS CORE
DEPARTMENT OF BIOSTATISTICS AND BIOINFORMATICS
AND GLOBAL HEALTH
DUKE UNIVERSITY
DURHAM, NC 27708

WALLER, LANCE A, PHD, MS, BS
PROFESSOR
DEPARTMENT OF BIOSTATISTICS AND BIOINFORMATICS
ROLLINS SCHOOL OF PUBLIC HEALTH
EMORY UNIVERSITY
ATLANTA, GA 30322

MAIL REVIEWER(S)

LINGURARU, MARIUS GEORGE, PHD, MA, MS, BS
PROFESSOR
915 NORTH CAROLINA AVE SE
WASHINGTON, DC 20003

SCIENTIFIC REVIEW OFFICER

BLOOM, MICHAEL L, PHD
SCIENTIFIC REVIEW OFFICER
CENTER FOR SCIENTIFIC REVIEW
NATIONAL INSTITUTES OF HEALTH
BETHESDA, MD 20892

EXTRAMURAL SUPPORT ASSISTANT

KIM, KIMI GAYUN
LEAD EXTRAMURAL SUPPORT ASSISTANT
CENTER FOR SCIENTIFIC REVIEW
NATIONAL INSTITUTES OF HEALTH
BETHESDA, MD 20892

Consultants are required to absent themselves from the room during the review of any application if their presence would constitute or appear to constitute a conflict of interest.
